# Modeling gene-environment interactions in longitudinal family studies: a comparison of methods and their application to the association between the IGF pathway and childhood obesity

**DOI:** 10.1186/s12881-018-0739-x

**Published:** 2019-01-11

**Authors:** Cheng Wang, Marie-Hélène Roy-Gagnon, Jean-François Lefebvre, Kelly M. Burkett, Lise Dubois

**Affiliations:** 1 School of Epidemiology and Public Health, University of Ottawa, 600 Peter Morand Cres, Ottawa, Ontario K1G 5Z3 Canada; 2 Department of Mathematics and Statistics, University of Ottawa, 150 Louis-Pasteur Pvt, Ottawa, Ontario K1N 6N5 Canada

**Keywords:** Childhood obesity, Insulin-like growth factors, Twin studies, Longitudinal family studies, Gene-environment interaction, Non-linear interaction, Simulation studies

## Abstract

**Background:**

The interactive effect of the IGF pathway genes with the environment may contribute to childhood obesity. Such gene-environment interactions can take on complex forms. Detecting those relationships using longitudinal family studies requires simultaneously accounting for correlations within individuals and families.

**Methods:**

We studied three methods for detecting interaction effects in longitudinal family studies. The twin model and the nonparametric partition-based score test utilized individual outcome averages, whereas the linear mixed model used all available longitudinal data points. Simulation experiments were performed to evaluate the methods’ power to detect different gene-environment interaction relationships. These methods were applied to the Quebec Newborn Twin Study data to test for interaction effects between the IGF pathway genes (IGF-1, IGFALS) and environmental factors (physical activity, daycare attendance and sleep duration) on body mass index outcomes.

**Results:**

For the simulated data, the twin model with the mean time summary statistic yielded good performance overall. Modelling an interaction as linear when the true model had a different relationship influenced power; for certain non-linear interactions, none of the three methods were effective. Our analysis of the IGF pathway genes showed suggestive association for the joint effect of IGF-1 variant at position 102,791,894 of chromosome 12 and physical activity. However, this association was not statistically significant after multiple testing correction.

**Conclusions:**

The analytical approaches considered in this study were not robust to different gene-environment interactions. Methodological innovations are needed to improve the current methods’ performances for detecting non-linear interactions. More studies are needed in order to better understand the IGF pathway’s role in childhood obesity development.

**Electronic supplementary material:**

The online version of this article (10.1186/s12881-018-0739-x) contains supplementary material, which is available to authorized users.

## Background

Childhood obesity is an important public health issue due to its negative impact on individual wellbeing and on the healthcare system [1–7]. To fully elucidate the complex processes underlying childhood obesity, we need to consider the joint gene-environmental basis of this disease. Studying gene-environment (GE) interactions can improve our understanding of how genetic differences contribute to phenotype variability in the population [8]. This is important for childhood obesity since currently identified genetic associations can only explain 1.45% of the body weight variation, while heritability studies have estimated that 40 to 70% of this variance is under genetic influence [9]. GE interactions likely play an important role in obesity development, as the disease is also attributed to many environmental causes. In addition to genetic predisposition, various environmental factors also affect energy intake and expenditure, the two primary driving forces behind obesity development. These environmental risk factors include high dietary sugar consumption, poor sleep habits, comfortable ambient temperature, low physical activity and large-scale social forces such as media influence [10–12]. An example of such interactions is that physical activity has been shown to modify the obesity risks associated with the FTO gene variants [9]. Thus, given the limitations in our understanding of childhood obesity, it is important to explore GE interactions to help us clarify the genetic basis of this disease.

The insulin-like growth factor (IGF) pathway genes, which are intimately involved in body fat regulation, have functional effects that can be influenced by the environment. The insulin-like growth factor-1 (IGF-1) proteins can directly modulate carbohydrate metabolism and adipose tissue growth [13, 14]. The growth hormone (GH) and the insulin-like growth factor binding protein, acid-labile subunits (IGFALS) regulate IGF-1 availability by increasing its production and circulation concentration, respectively [13–18]. The effect of those proteins can be modified by many environmental factors. Increased physical activity has been shown to be associated with higher GH and IGF-1 levels [19–21]. GH production in the body also correlates with the length of the slow wave phase of sleep [22]. Indirectly, the IGF pathway and daycare attendance may interact via inflammation-related neutrophil proteases that target the IGF proteins [23]. This is because children attending daycare facilities are at increased risk for infectious diseases, and consequently more frequent occurrence of inflammatory response [24]. For example, the proportion of children with common childhood illnesses like lower respiratory infections and diarrhea is significantly higher in children who attend daycare. There is also some evidence that children attending daycare are at increased risk of obesity [25–27]. Given the IGF pathway’s involvement in body fat regulation, environmental modulations of the pathway could manifest as GE interaction effect on obesity development. Thus in this study, we hypothesize three potential GE interaction scenarios between the IGF pathway genes (IGF-1, IGFALS) and environmental factors (physical activity, daycare attendance and sleep duration).

One way to evaluate potential GE interactions is by conducting longitudinal family studies. This involves recruiting samples of related individuals, and collecting data repeatedly on the same participant over time. Longitudinal family data can provide additional information and increased statistical power, but at the cost of analytical challenges due to correlated data structure. The longitudinal aspect of the study can provide information relating to the temporal pattern of genetic contributions to a trait, while knowledge of family structure can help in imputing missing genotype data and determining segregation patterns [28, 29]. Improvement in statistical power comes from the fact that more reliable data are available through repeated measurement, and the ability to sample families rich in potentially associated genetic variants [29, 30]. However, leveraging the benefits of longitudinal family studies requires properly accounting for correlations due to familial relations and repeated outcomes in data analysis. One approach involves incorporating random effects with an assumed covariance structure to accommodate both familial and over time correlations. This can be achieved in the linear mixed model framework [31, 32]. Alternatively, simplified correlation structure allows the application of models not designed for longitudinal family data. Repeated outcomes can be transformed into summary statistics (e.g. mean over time) and analyzed using cross sectional methods like the twin study model [33]. Familial correlation can be simplified by ignoring the difference in correlations due to different kinship relations within a family. The family-wise permutation test for *p*-value estimation represents one such approach. This permutation procedure accounts for familial correlation by restricting the random shuffling of outcome-predictor pairing to each family, but without distinguishing the specific pedigree relations within the family [34]. However, model simplification by averaging over time or assuming equal familial correlation could result in a loss of power as all the information in the data is not being utilized.

In addition to the complex data structures encountered in longitudinal family studies, GE interaction relationships can take on many forms. This creates difficulty when modeling the interaction effect parametrically, as the effect pattern can be misspecified. A linear interaction pattern is assumed when modeling with the conventional multiplicative product term in regression models. [35] However, many non-linear effect patterns are also possible. For example, genetic and environmental factors may interact in an extremely non-linear manner called exclusive OR (XOR) interactions. This can be interpreted as two opposing patterns of genetic effect under different environmental conditions. An example is the case of sickle cell anemia, where having one copy of the disease allele confers improved survival if the individual lives in malaria affected regions. Similar effects are also observed in psychiatric genetics in the case of plasticity genes. Individuals with certain alleles are hypothesized to be more receptive to environmental factors that may be either beneficial or harmful depending on the specific environmental conditions [36]. For instance, individuals with low-activity variant of the monoamine oxidase-A (MAOA) gene have been reported to be both more and less antisocial depending whether they are exposed to childhood maltreatment [36]. In addition to XOR relations, GE interaction effect could manifest in a conditional dominant fashion, where an additive pattern of genetic effect is observed when the environmental exposure is absent. But, when the environmental exposure is present, the genetic effect is enhanced and immediately plateaus, producing a dominant pattern of inheritance. A third type of non-linear GE relation is the small marginal effect interaction. This GE interaction is the most difficult to detect because modest genetic effect (e.g. effects produced by recessive pattern of inheritance) will be triggered only when an environmental exposure is present.

It is clear that using regression product terms to model interactions is only theoretically appropriate when the interaction pattern is linear, and hence this is a strong assumption that may not be suitable for non-linear scenarios. Nonparametric approaches have been developed to relax this assumption. For example, the partition-based score I (PBI) test evaluates hypothesized interactions between categorical factors by comparing data sets partitioned using different combinations of predictor variables [34, 35]. The significance of interactions is assessed by comparing the outcome explanatory powers of different partitioning variable combinations, and so the test does not assume specific interaction relationships between the factors [34, 35].

In this study, we evaluated potential GE interactions between the IGF pathway genes (IGF-1, IGFALS) and environmental factors (physical activity, sleep duration and daycare attendance) in the context of a longitudinal family study. Data were obtained from the Quebec Newborn Twin Study (QNTS) which followed a birth cohort comprised of twin pairs from Montreal, Canada, who were born between 1995 and 1998 [37]. A variety of social, biological and psychological measures on the QNTS participants were obtained from shortly after birth to early adolescent years [37, 38]. As the analysis strategy for this type of study is unclear due to complex longitudinal-familial correlations and non-linear interactions, we first simulated data based on the characteristics of the QNTS participants, and systematically evaluated the performances of three analytical methods (the linear mixed model, the twin study model and the PBI test) for detecting different GE interaction relations in longitudinal family data. Specifically, we assessed model performances with respect to the effect of simplifying correlation structures and their robustness under non-linear interaction scenarios. Next, we applied the considered models to test for potential IGF pathway GE interaction effect on body mass index (BMI) measured longitudinally in QNTS.

## Methods

### The Quebec newborn twin study

Data for this study were obtained from the Quebec Newborn Twin Study (QNTS) which followed twin pairs recruited from Montreal, Canada, who were born between 1995 and 1998 [37]. The studied population excluded twins with major illness at birth or those who died before 5 months of age [38, 39]. Zygosity was determined using a multitude of evidence including chorionicity data, physical similarity as well as genotype data [37, 39]. QNTS collects data on a variety of social, biological and psychological measures through medical records, interviews, and laboratory assessments. The follow-up assessment is still ongoing with longitudinal data available from shortly after birth and to early adolescent years [37, 38]. Separate data collection personnel were assigned for each individual within a twin pair to control for bias due to knowledge of zygosity status [37]. Details on the QNTS study can be found in Boivin et al. [37]. The overall structure of QNTS data consists of repeated measures for individuals nested within families (twin pairs).

### Statistical analysis

We investigated three different analytical methods for longitudinal family studies. Each method uses a combination of different approaches for modeling correlation structure and interaction relationships, as outlined below. All analyses were performed using R [40].

#### Twin model

The twin model refers to the path model used in classical twin studies [41]. The model equation is formulated as follows:

$$ {Y}_{ij}={\boldsymbol{x}}_{\boldsymbol{ij}}\boldsymbol{\beta} +a{A}_{ij}+c{C}_{ij}+e{E}_{ij} $$

*Y*_*ij*_ represents the BMI outcome for twin pair *i*, individual *j*. ***x***_***ij***_***β*** is the systematic component of the model, which includes the design matrix predictor values (***x***_***ij***_) and its associated fixed effects (***β***). The random variation in the outcome is modeled by *A*_*ij*_, *C*_*ij*_, and *E*_*ij*_ terms, which are mutually independent standard normal random variables. The *a*, *c*, *e* terms are the pathway coefficients that reflect the partitioning of the outcome variance due to background additive genetic effect (*a*), common environment influence (*c*) and random environment variation (*e*). Each of the variance components is equal to its path coefficient squared. We used the *twinlm* function from the *mets* R package to fit the model [42].

In order to analyze repeated measures using the twin model, we summarized the outcome measures (BMI) for each individual by their averages across the time points. The variance partitioning setup of the model is able to accurately account for the difference in correlations between MZ and DZ twins as described in the introduction. However, it is not currently possible to model the correlation due to longitudinal measurements in the twin model. Since the systematic component of the twin model is formulated as a typical linear regression model, it will assume a linear interaction relationship by using a multiplicative term for the interaction effect. In addition to an additive genetic model (0, 1, 2 for the number of minor alleles), we also used a co-dominant specification for the genetic effect. Under a co-dominant model, heterozygotes and minor allele homozygotes were represented using separate indicator variables with the common allele homozygotes as the reference category. This allows for a more flexible interaction model.

#### Linear mixed model

The linear mixed model approach considered in this study was implemented using the *lmekin* function from the *coxme* R package [43]. The linear mixed model utilizes all of the repeated measures and models longitudinal correlation via individual random effect [44]. Each individual receives a random intercept to induce correlation among his/her repeated outcomes. The correlation due to different family structure is accounted for by using a kinship matrix to specify the covariance structure of the random effect. The entries in the kinship matrix are kinship coefficients that reflect genetic relatedness between individuals. These kinship coefficients will specify the covariance of individual random effect with self and with others (See Section S1, Additional file 1 for details on kinship matrix construction). The linear mixed model is a regression-based method and will implicitly assume linear interaction when using multiplicative term to represent GE interaction effect. As with the twin model, we specified the genetic effect using additive and co-dominant coding. Interactions between genotype status and the environmental variable were modeled using multiplicative terms. *P*-values for the fixed effects were based on a Wald test or a likelihood ratio test if the effect was coded using multiple indicator variables.

#### Partition-based score I test

The partition-based score I (PBI) test is a nonparametric approach for assessing interactions by contrasting the outcome variations explained under different ways of partitioning the dataset, with each induced by considering different subsets of the categorical predictor variables [35]. For a given partition scheme, the dataset is split into sub-datasets according to the levels of the considered predictor variables. Thus, a portion of the variation in the outcome will be explained by the predictor variables that specify the partitioning. A dispersion statistic that measures the amount of the explained outcome variation by a set of partitioning variables is estimated as follows:

$$ I=\sum \limits_{i=1}^k\frac{n_i}{n}\bullet \frac{{\left({\overline{y}}_i-\overline{y}\right)}^2}{\raisebox{1ex}{${s}_y^2$}\!\left/ \!\raisebox{-1ex}{${n}_i$}\right.} $$

The sub-datasets generated from the partitioning process is denoted by *i*, where *i* = 1, 2…*k*. ${\overline{y}}_i$ is the mean outcome for the *i*^*th*^ partitioned sub-dataset. *n*_*i*_ is the sample size for the *i*^*th*^ sub-dataset. $\overline{y}$ and ${S}_y^2$ are overall outcome mean and sample variance respectively. *n* is the overall sample size.

The PBI test statistic is estimated as the difference between the dispersion statistics obtained from partitioning using both interacting variables and the maximum of the dispersion statistics estimated when partitioning by each variable alone. It is formularized as below:

$$ {I}_T={I}_{GE}-\max \left({I}_{G,}{I}_E\right) $$

To evaluate a potential GE interaction, we estimate the dispersion statistics for a dataset that is partitioned by both genetic and environmental variables (*I*_*GE*_) and by each variable separately (*I*_*G*_, *I*_*E*_). The test statistic (*I*_*T*_) in this case is the difference between *I*_*GE*_ and the maximum of *I*_*G*_, *I*_*E*_. The *p*-value of the test is estimated using permutation. The permutation procedure accounts for familial correlation by constraining the permutation step to within each family. In this study, the test p-value was estimated with 10,000 permutation replications.

The PBI test is not designed to utilize repeated outcome measures. In order to analyze longitudinal data using the PBI test, we use the average BMI over time as a summary measure for each individual. To allow dataset division, the predictors specifying a given partition scheme must be categorical. Any continuous predictors would need to be categorized before applying the test. The PBI test is nonparametric and does not assume linear interaction relationship.

### Simulation analysis

Simulated BMI trajectories for 788 individuals (226 DZ twins, 168 MZ twins) were generated with input predictors that were partially based on their corresponding QNTS variables. Actual QNTS data on the individual’s family structure, zygosity, sex and age were used for the simulation input. Missing data on age were imputed by randomly sampling the age distribution of the whole sample at the missing time point.

Hypothetical genetic and environmental factor data were generated using probability models. The environmental factor was coded as either present (1) or absent (0), and was sampled from a Bernoulli distribution independently for each individual with exposure frequency set to 0.3. The genetic factor was coded to reflect additive genetic effect (0, 1, and 2 for the number of minor alleles). Hypothetical parental genotypes for each individual were generated as the sum of two independent Bernoulli random variables, thus assuming random mating. The minor allele frequency was set to 0.3. Individual genotype data was then obtained from parental genotypes using probabilities according to Mendelian inheritance pattern.

**Fig. 1:**
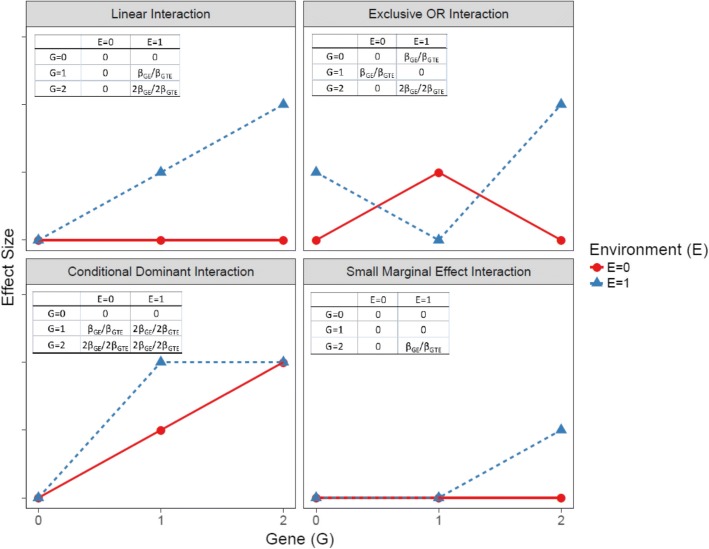
Effect specification for various interaction scenarios. β_GE_ and β_GTE_ are interaction effect parameters for gene-environment and gene-time-environment interactions respectively. Genetic (G) factor coded as 0, 1, and 2 for the number of minor alleles. Environment (E) factor coded as 1 or 0 for presence or absence of the environmental exposure. The tables in each panel present the interaction effect θ_ijk_ associated with each combination of the genetic (G) and environmental (E) factors

Longitudinal BMI data trajectories were simulated using a linear mixed model. The model equation is formulated as follow:

$$ {Y}_{ij k}=\left({\beta}_0+{C}_{0 ij}\right)+{\beta}_S{S}_{ij}+\left({\beta}_T+{C}_{Tij}\right){T}_{ij k}+{\beta}_{T^{\prime }}{T}_{ij k}^{\prime }+{\beta}_G{G}_{ij}+{\beta}_{GT}{G}_{ij}\times {T}_{ij k}+{\beta}_E{E}_{ij}+{\theta}_{ij k}+\varepsilon $$

$$ where\kern0.5em \theta =\left\{{}_{\beta_{GT E}h\left({G}_{ij},{T}_{ij k},{E}_{ij}\right),\kern0.5em if\kern0.5em GTE\kern0.5em \mathrm{interactions}}^{\beta_{GT}f\left({G}_{ij},{E}_{ij}\right),\kern0.5em if\kern0.5em GE\kern0.5em \mathrm{interactions}}\right. $$

and functions *f*( ) and *h*( ) assign individual covariate values for the interaction effect according to the specified interaction scenarios (Fig. 1).

*θ*_*ijk*_ determines the simulated GE interaction effect on the outcome for each individual at each time point. The effect pattern is specified using an interaction matrix describing the combined interactive effect of the genetic factor and the environmental factor (Fig. 1). We simulated scenarios corresponding to the four interaction relationships discussed in the introduction. *β*_*GE*_ and *β*_*GTE*_ represent the interaction effect between gene and environment (*GE*) and gene-time-environment (*GTE*) respectively.

*Y*_*ijk*_ is the BMI trait value for individual *j* of the *i*^*th*^ twin pair at time point *k*. *β*^′^*s* with the corresponding subscripts represents fixed effects for age in months (*T*_*ijk*_), sex (*S*_*ij*_), the genetic factor (*G*_*ij*_) and the environmental factor (*E*_*ij*_). *β*_*GT*_ specifies the linear interaction effect between the genetic factor (*G*_*ij*_) and time (*T*_*ij*_).

${\beta}_{T^{\prime }}T{\prime}_{ijk}$ is used to modify the rate of change in BMI over time to allow simulation of a segmented longitudinal trend for BMI that resembles the actual trajectories found in QNTS. Specifically, the *T*′_*ijk*_ value is assigned as follows:

$$ T{\prime}_{ijk}=\left\{\begin{array}{c}0, if\ k\le 2\\ {}{T}_{ijk}-6, otherwise\end{array}\right. $$

Based on this specification, an additional time effect from ${\beta}_{T^{\prime }}$ is applied after 6 months of age (time point, *k* > 2). This replicates the initial phases of fast growth before 6 months and the subsequent plateauing of BMI afterwards as observed in QNTS data.

*C*_*oij*_ and *C*_*Tij*_are the random effects on the intercept and the rate of change of BMI over time, respectively, for each individual. The effects are simulated as components of a bivariate normal random vector for each twin pair. The covariance structure of the random vector is set up to reflect the difference in familial correlation between MZ and DZ twins due to background additive genetic effect.

$$ {C}_{0 ij}\sim N\left(0,{V}_0\right),{V}_0=\left[\begin{array}{cc}{\sigma}_A^2+{\sigma}_C^2& \left\{\begin{array}{c}{\sigma}_A^2+{\sigma}_C^2\  if\  MZ\\ {}\frac{1}{2}{\sigma}_A^2+{\sigma}_C^2\  if\  DZ\end{array}\right.\\ {}\left\{\begin{array}{c}{\sigma}_A^2+{\sigma}_C^2\  if\  MZ\\ {}\frac{1}{2}{\sigma}_A^2+{\sigma}_C^2\  if\  DZ\end{array}\right.& {\sigma}_A^2+{\sigma}_C^2\end{array}\right] $$

$$ {C}_{Tij}\sim N\left(0,{V}_T\right),{V}_T=\left[\begin{array}{cc}{\tau}_A^2+{\tau}_C^2& \left\{\begin{array}{c}{\tau}_A^2+{\tau}_C^2\  if\  MZ\\ {}\frac{1}{2}{\tau}_A^2+{\tau}_C^2\  if\  DZ\end{array}\right.\\ {}\left\{\begin{array}{c}{\tau}_A^2+{\tau}_C^2\  if\  MZ\\ {}\frac{1}{2}{\tau}_A^2+{\tau}_C^2\  if\  DZ\end{array}\right.& {\tau}_A^2+{\tau}_C^2\end{array}\right] $$

Parameters ${\sigma}_A^2$ and ${\tau}_A^2$ represent the additive genetic effect on BMI correlation while ${\sigma}_C^2$ and ${\tau}_A^2$ specify the common environmental effect on the correlation.

Simulation model parameters for genetic and environmental factors were chosen to discern potential power differences between the analytic models. Parameter values for other variables were based on actual QNTS data. The scenarios were broadly categorized into models with no interaction effect, interaction effect on the outcome average and interaction effect on the outcome rate of change over time. For the no interaction effect scenarios, we varied the genetic and the environment effects that were independent of each other. To estimate power for detecting GE interactions, we varied the interaction effect size (*β*_*GTE*_ and *β*_*GE*_). See Table 1 for details on simulation parameterization.

**Table 1:** Parameter configuration for simulation scenarios

Parameter^b^	Gene-environment interaction effect modeled^a^					
	No Effect		Effect on Average		Effect on Change	
	No Effect on Average^c^	No Effect on Change^c^	Linear Interaction	Non-linear Interaction^d^	Linear Interaction	Non-linear Interaction^d^
β_0_	11	11	11	11	11	11
β_S_	0.5	0.5	0.5	0.5	0.5	0.5
β_T_	0.8	0.8	0.8	0.8	0.8	0.8
β_T’_	−0.8	−0.8	− 0.8	−0.8	− 0.8	−0.8
β_G_	0 to 1	0	0.25	0	0	0
β_GT_	0	0 to 0.03	0	0	0.01	0
β_E_	0 to 2	0 to 1.2	0.5	0	0.5	0
σ^2^_A_	3	3	3	3	3	3
σ^2^_C_	1.5	1.5	1.5	1.5	1.5	1.5
τ^2^_A_	0.001	0.001	0.001	0.001	0.001	0.001
τ^2^_C_	0.001	0.001	0.001	0.001	0.001	0.001
σ^2^_E_	1.5	1.5	1.5	1.5	1.5	1.5
β_GE_	0	0	0.1 to 1	0.1 to 1	0	0
β_GTE_	0	0	0	0	0.001 to 0.03	0.001 to 0.03

^a^A range of values was simulated for some parameters, effect sizes varied at 0.1 increments for β_GE_ when the interaction effect was on the average. For interaction effect on the rate of change scenarios, β_GTE_ varied at an increment of 0.003 before reaching 0.01 and then by 0.005 afterwards. Under no interaction effect scenarios, β_G_ varied at the same increments as β_GE_ (no effect on average), and β_GT_ varied at the same increments as β_GTE_ (no effect on change). β_E_ varied at 0.2 increments (no effect on average) or was inflated by 40 times relative to its corresponding β_GT_ value (no effect on change)

^b^β_0_: average BMI at baseline predictor level; β_S_: sex effect; β_T_, β_T’_: time effect; β_G_: genetic effect; β_GT_: gene-time (GT) interaction effect; β_E_: environmental effect; σ^2^_A_: additive genetic effect on random intercept correlation; σ^2^_C_: common environmental effect on random intercept correlation; τ^2^_A_: additive genetic effect on random slope correlation; τ^2^_C_: common environmental effect on random slope correlation; σ^2^_E_: common effect on BMI variance; β_GE_: gene-environment (GE) interaction effect; β_GTE_: gene-time-environment (GTE) interaction effect

^c^For no interaction effect models, the scenarios refer to the trends where there are no GE interaction effect on genetic main effect (no effect on average) or gene-time interaction effect (no effect on change)

^d^Non-linear interaction scenarios included exclusive OR (XOR) interaction, conditional dominant interaction and small marginal effect interaction

For each simulation scenario, we applied the analytical models to 2000 simulated dataset replicates. The power of each analysis method to detect GE interactions was estimated as the proportion of significant results (*p*-value < 0.05) over the 2000 replicates. Significance tests were performed on the interaction terms (*β*_*GE*_ and *β*_*GTE*_) for regression-based models and on the PBI test statistic. For null interaction scenarios, the proportion of significant results (p-value < 0.05) was used to estimate the type 1 error rate. For the twin and the linear mixed models, we adjusted for sex, age, gene and environment main effects in the model.

### IGF pathway GE interaction analysis

To assess GE interactions between the IGF pathway genes (IGF-1, IGFALS) and environmental factors (physical activity, sleep duration and daycare attendance), we analyzed actual QNTS data on a sample of 536 individuals from 292 families (143 MZ twins, 149 DZ twins). The initial sample had 810 individuals with available environmental data, in which 682 individuals also had sequencing data. Details on the analysis sample selection are in Section S1, Additional File 1.

Exon Sequencing data on the IGF-1 and IGFALS genes were obtained using the Illumina HiSeq 2500 platform at McGill University and Génome Québec Innovation Centre. Molecular Inversion Probe (MIP) method was used to construct DNA libraries for which deep sequencing was carried out. We focused our analysis on common single nucleotide polymorphism (SNP) variants and excluded those whose minor allele frequencies (MAF) were less than 0.05. Data quality control exclusions including sequencing quality, missing data proportion (> 10%) and Hardy-Weinberg Equilibrium (HWE) test (*p*-value < 0.01) were also applied. SNPs with highest MAF were selected from haplotypes that are in linkage disequilibrium (R^2^ > 0.8). The final analysis was performed on 7 SNPs from the IGF-1 gene and 2 SNPs from the IGFALS gene. Sequencing data was coded according to the additive genetic effect model where the number of the minor alleles is counted (0, 1, and 2).

BMI data were obtained for each individual at 6 time points from birth to around 6 years of age. Data were collected using medical record (at birth), laboratory assessment (6 month and 62 month follow-up) and interview (all other follow-up) [38]. 99 individuals were excluded for missing more than 4 out of the 6 BMI measurements. We also filtered out 6 BMI measures from 6 individuals that were judged to be unrealistically high/low and likely measurement or recording errors. BMI data was treated as a continuous variable without any recoding.

Zygosity status was coded dichotomously as either 0 (DZ twins) or 1 (MZ twins). Individual age was recorded as number of months allowing decimals to account for partial month. We did not differentiate between different gestational ages among individuals, and set all ages to be zero at birth. Sex was coded as binary with 0 being female and 1 being male.

Interviews were conducted at the follow-up time points to obtain information on environmental exposures. We collapsed them into a single summary measure per individual, as described below. Physical activity level was assessed only at 32 and 50 months follow-up where parents were asked to rate the physical activity level of the study participant relative to his/her peers of same age and sex. Since the majority of responses remained unchanged between the two assessments, we only used the 32-month follow-up data in our analysis as it is the midpoint of the follow-up period. The responses were collapsed into a 3-level categorical variable (“more or a lot more”, “equal” and “less or a lot less”) with the most active category as the baseline. Extreme categories were combined due to low counts.

Information on daycare attendance was obtained through parental interview at 4 time points (6, 20, 32, 50 months of age). Attendance status was assessed by asking the parents whether the participant was using daycare or other babysitting services including care by relative at the time of interview. We summarized the overall daycare attendance for an individual by the proportion of his/her follow-up time where the individual attended daycare service. If the response was “yes” at any given time point, we assumed that the participants attended daycare service for the full duration of time until the next follow-up point. Attendance status was excluded if we could not calculate the time between two follow-up points due to missing data for age. The analysis variable was a continuous proportion ranging from 0 to 1.

Individual sleep time was measured by two parental interview questions on day and night sleep times, in which parents were asked to describe the amount of time the participants slept during day and night time. An individual’s overall sleep time at each time point was calculated as the sum of his/her day and night sleep durations in hours (See Section S1, Additional file 1 for details on deriving total sleep time data). We then scored each individual on whether his/her total sleep time met the minimum recommended level from the American Academy of Sleep Medicine (See Section S1, Additional file 1) and endorsed by the American Academy of Pediatrics [45, 46]. Finally, since sleep time data were available for 4 follow-up time points (6, 20, 32, 50 months of age), we summarized individual sleep duration status as the proportion of follow-up time where the subject’s total sleep time met the minimum recommended level. Follow-up proportion was calculated similarly as daycare service attendance.

We used individual’s reported race as a measure of their ethnic background. 8% of the responses did not consider themselves to be Caucasian (“white”). The MAF for the IGF-1 and IGFALS SNPs were markedly different for Caucasian and non-Caucasian individuals (See Section S1, Additional file 1). We thus excluded 47 individuals with non-Caucasian ethnicity from our analysis in order to control for confounding due to ethnicity (population stratification).

Univariate analyses were conducted to obtain sample distributions for the variables. We compared the distributions of environmental exposures and other covariates between the filtered sample used in our analysis and the initial QNTS data sample. Depending on the variable type, Chi-squared test or ANOVA test were applied to BMI, age, zygosity, sex, physical activity, daycare attendance, sleep duration and race. The previously described twin model and the linear mixed model were fit to assess potential GE interactions between each SNP-environmental factor pair. The interaction was modeled with a multiplicative term in the regression equation. Both the twin model and the linear mixed model adjusted for sex in addition to genetic and environmental predictors. The time effect was modeled as a segmented trend in the linear mixed model with a knot placed at age = 6 months (approximately the 2nd follow-up time point). The PBI test was performed to evaluate interaction effects between each SNP-environmental factor pair. Continuous environmental exposures were categorized according to their quartile levels to allow dataset partitioning with the PBI test. For each method, Bonferroni multiple comparison adjustment was made for GE interaction tests involving the same environmental exposure (number of tests = 9). Goodness of fit for the twin model and the linear mixed model was assessed by their residual plots. Sensitivity analyses were conducted to assess the effect of excluding impossible BMI values and non-Caucasian individuals on our conclusions.

## Results

### Detecting GE interactions in longitudinal family data

We simulated two sets of scenarios, where the interaction effect was either on the average or on the rate of change in BMI over time. In this section, we present results from the additive genetic effect coding for the linear mixed model and the twin model. The additive and co-dominant model specifications yielded qualitatively similar results and thus similar conclusions regarding the comparison of the analytical methods. Results from the analysis using the co-dominant specification are shown in Supplementary Section S2.

**Fig. 2:**
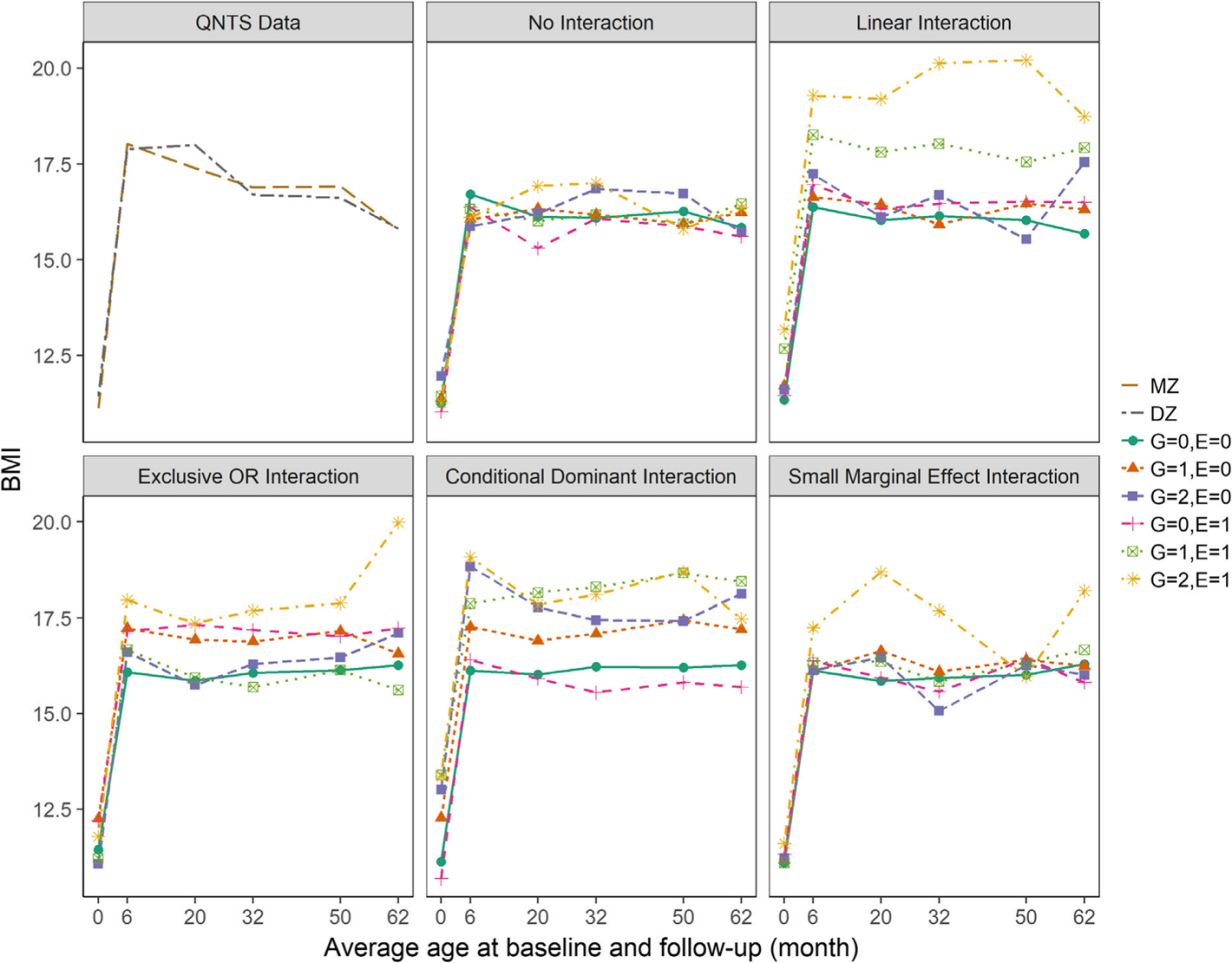
Trajectories of average BMI over time for actual QNTS data and simulated gene-environment interaction scenarios. Example datasets from each simulation scenario having an interaction effect on the BMI average are compared to the actual QNTS data. Trajectories of average BMI at each time point based on actual QNTS data are grouped by zygosity status; MZ (monozygotic twin) and DZ (dizygotic twin). Averages of simulated BMI at each time point and their trajectories are grouped by genetic (G) and environmental (E) factor levels. Genetic factor coded as 0, 1, and 2 for the number of minor alleles. Environment factor coded as 1 or 0 for presence or absence of the environmental exposure. Simulated data generated for both interaction effect on the average scenarios and no interaction effect null scenarios

**Fig. 3:**
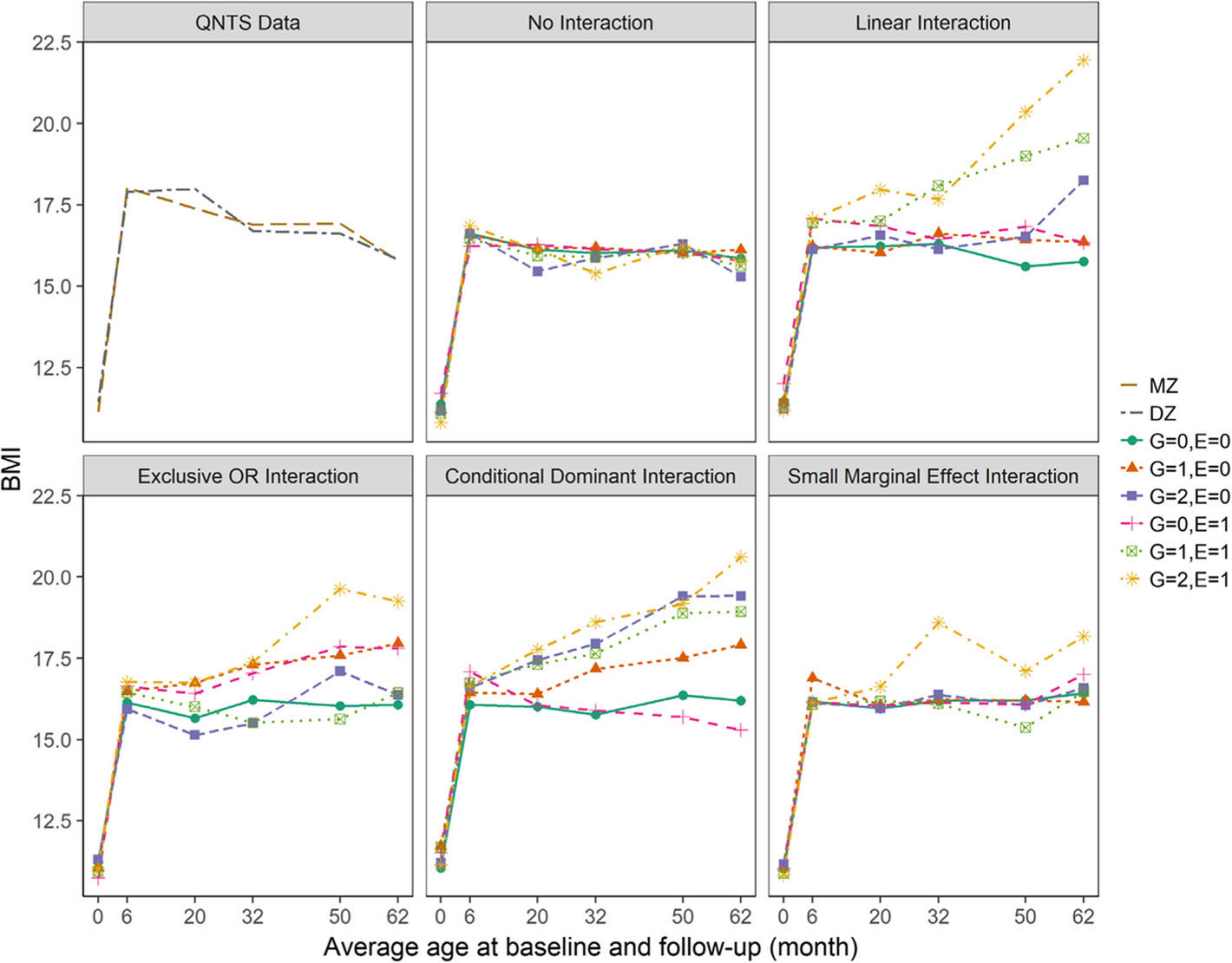
Trajectories of average BMI over time for actual QNTS data and simulated gene-time-environment interaction scenarios. Example datasets from each simulation scenario having an interaction effect on the rate of change of BMI over time are compared to the actual QNTS data. Trajectories of average BMI at each time point based on actual QNTS data are grouped by zygosity status; MZ (monozygotic twin) and DZ (dizygotic twin). Averages of simulated BMI at each time point and their trajectories are grouped by genetic (G) and environmental (E) factor levels. Genetic factor coded as 0, 1, and 2 for the number of minor alleles. Environment factor coded as 1 or 0 for presence or absence of the environmental exposure. Simulated data generated for both interaction effect on the rate of change over time scenarios and no interaction effect null scenarios

Figures 2 and 3 show average BMI trajectories from the QNTS data compared to those from one simulated dataset from each GE and GTE interaction scenario. Compared to the actual QNTS trajectories, simulated trajectories were in the same general range for BMI values. Differences in the averages between the simulated trajectories for each gene-environment factor level demonstrated the intended interaction effect for each simulation scenario.

**Fig. 4:**
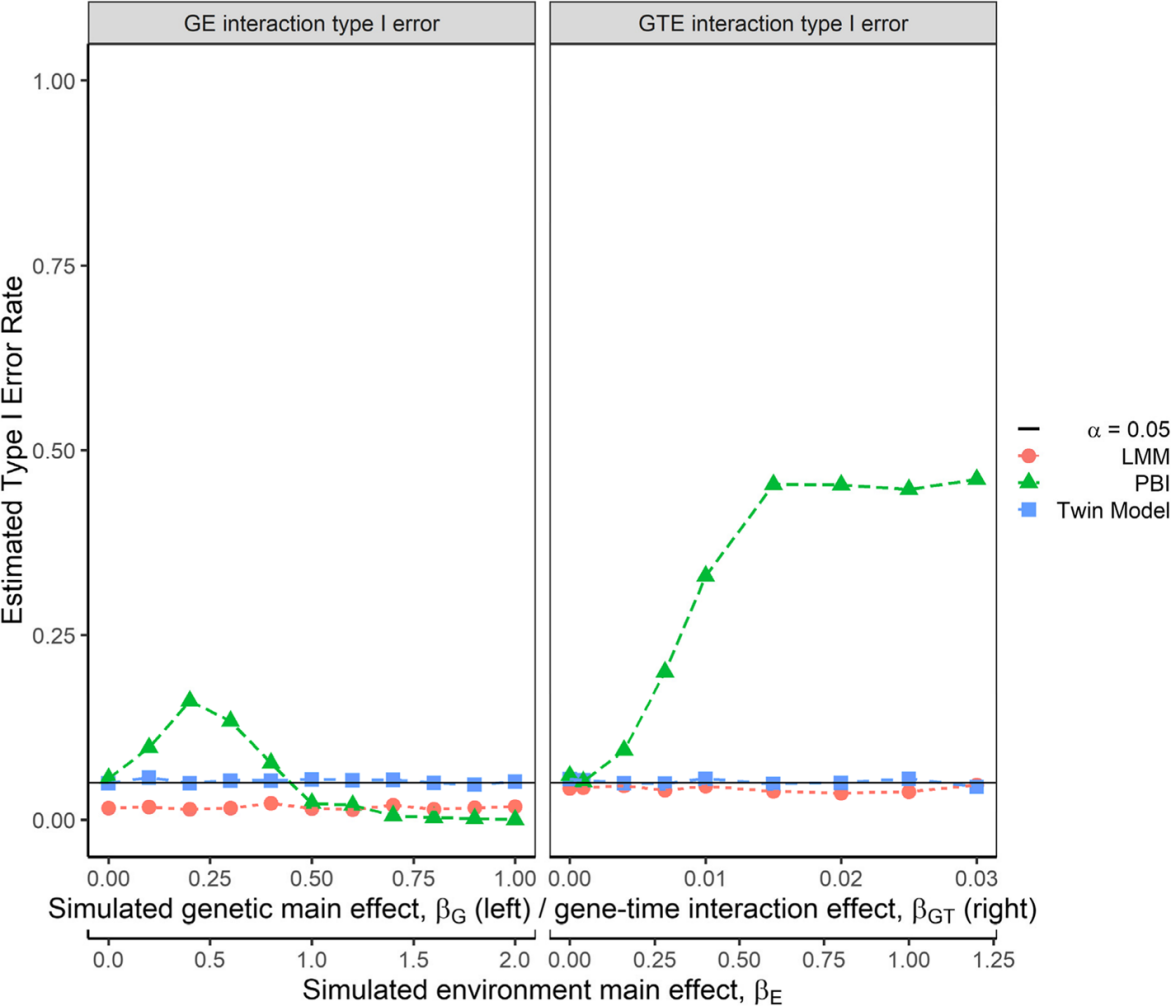
Estimated type I error rates for the compared analytical approaches. Type I error rates were estimated for each analytical approach as the proportion of false positive results (*p*-value < 0.05) calculated over 2000 simulation replicates with no interaction effect. Estimated type I error rates are shown for gene-environment (GE) interaction effect (left panel; simulation model includes a genetic main effect) and gene-time-environment (GTE) interaction effect (right panel; simulation model includes a gene-time interaction effect). The environmental main effect (β_E_) was included for all null scenarios

Figure 4 shows the estimated type 1 error rates for simulation scenarios without interaction effects. Both the twin and linear mixed models controlled the type 1 error rate well (estimated type 1 error rate < 0.05). The linear mixed model appeared to be more conservative in comparison to the other tests. The PBI test met the 0.05 threshold only when there was no main effect simulated. As the magnitude of genetic and environmental main effects increased in the null GE interaction scenario, the PBI test type I error rate exceeded 0.05 briefly (*β*_*G*_ from 0.1 to 0.4 and *β*_*E*_from 0.2 to 0.8). At higher main effect sizes (*β*_*G*_ > 0.4 and *β*_*E*_ > 0.8), the false positive rate decreased, and the test became more conservative in those scenarios. For the null GTE interaction scenario, the PBI test’s estimated type 1 error rate increased as the GT interaction and the environmental factor effects increased. This increase in false positives plateaued when *β*_*GT*_ = 0.015 and *β*_*E*_ = 0.6, and stabilized at a false positive rate of around 0.5.

**Fig. 5:**
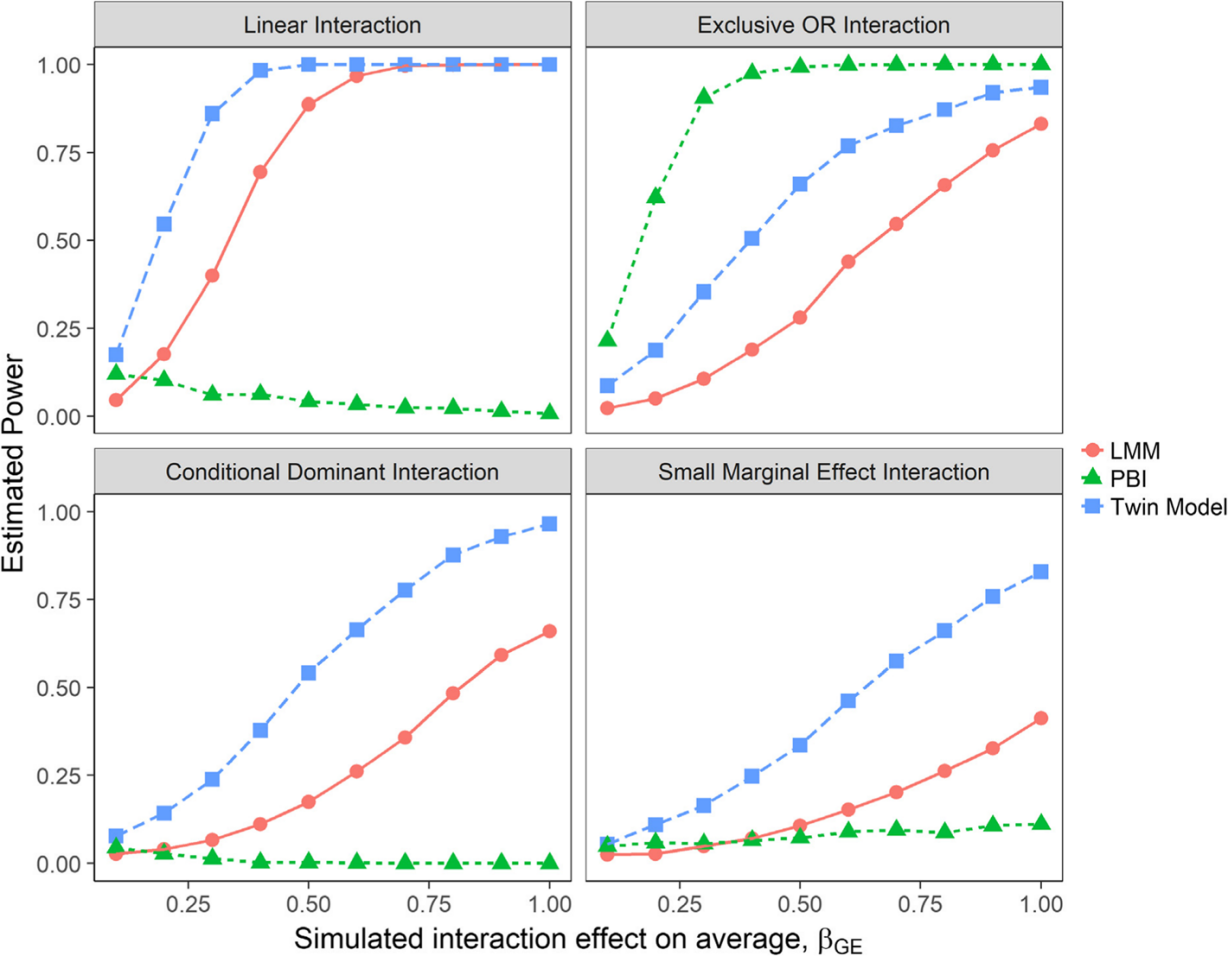
Estimated power to detect GE interaction effect on the average scenarios. Power was estimated for each analytical approach as the proportion of true positive result (p-value < 0.05) over 2000 simulation replicates for the considered interaction scenarios. LMM = linear mixed model; PBI = partition based score I test

The twin model was more powerful compared to the linear mixed model and the PBI test, except when the interactive relationship was extremely non-linear (Exclusive OR interaction or XOR) (Fig. 5). When the interactive relationship was XOR, the PBI test had the best performance. For linear interaction scenarios, regression-based models (twin and linear mixed models) performed well, while the PBI test had almost no power even at larger interaction effect sizes. All methods had lower power under small marginal effect and conditional dominant interaction scenarios, when compared with their respective best case scenarios with highest power.

**Fig. 6:**
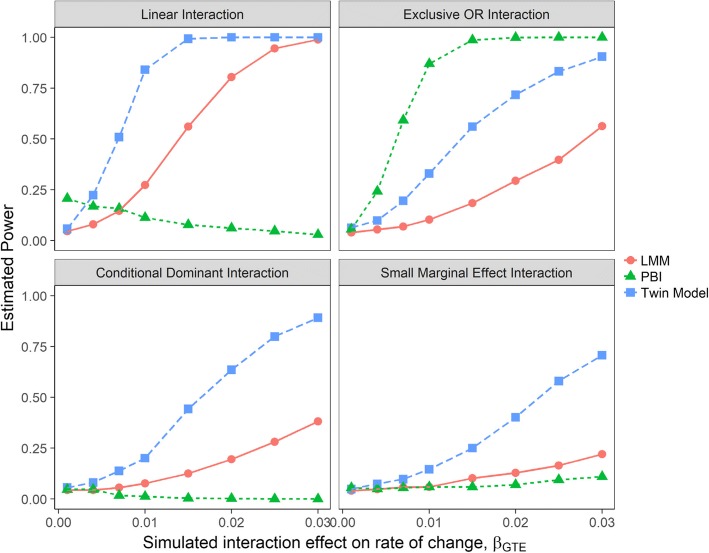
Estimated power to detect GTE interaction effect on the rate of change over time scenarios. Power was estimated for each analytical approach as the proportion of true positive result (p-value < 0.05) over 2000 simulation replicates for the considered interaction scenarios. LMM = linear mixed model; PBI = partition based score I test

The power analysis results for scenarios where the interaction effect acted on the rate of change were similar to the scenarios where the interaction effect acted on the average (Fig. 6). Regression-based methods (twin and linear mixed models) performed well for linear interaction situations (top right panel), but were less powerful for non-linear scenarios (other panels). The PBI test was powerful only when the relationship was significantly non-linear (XOR). The twin model had higher power compared to the PBI test and the linear mixed model except when the interaction was XOR. For the conditional dominant and small marginal effect interaction scenarios, all three methods had more difficulty detecting the true effects when contrasted with their respective best case scenarios.

From our simulation analysis, we observed unusual behavior for the PBI test. The test was either too conservative or very prone to false positives under different scenarios. Since the PBI test statistic is dependent on the dispersion statistic values (I_GE_, I_E_ and E_G_) calculated for different ways of partitioning the dataset, we monitored the values of those statistics for different simulation parameter values. In scenarios where the PBI test behaved erratically, partitioning the dataset by either genetic or environmental factor alone achieved comparable or higher outcome explanatory performance than partitioning by both gene-environment variables. Subsequently this affected the PBI test statistic and its significance. Detailed results can be found in Section S2, Additional file 1.

### IGF pathway GE interaction effect on BMI

**Fig. 7:**
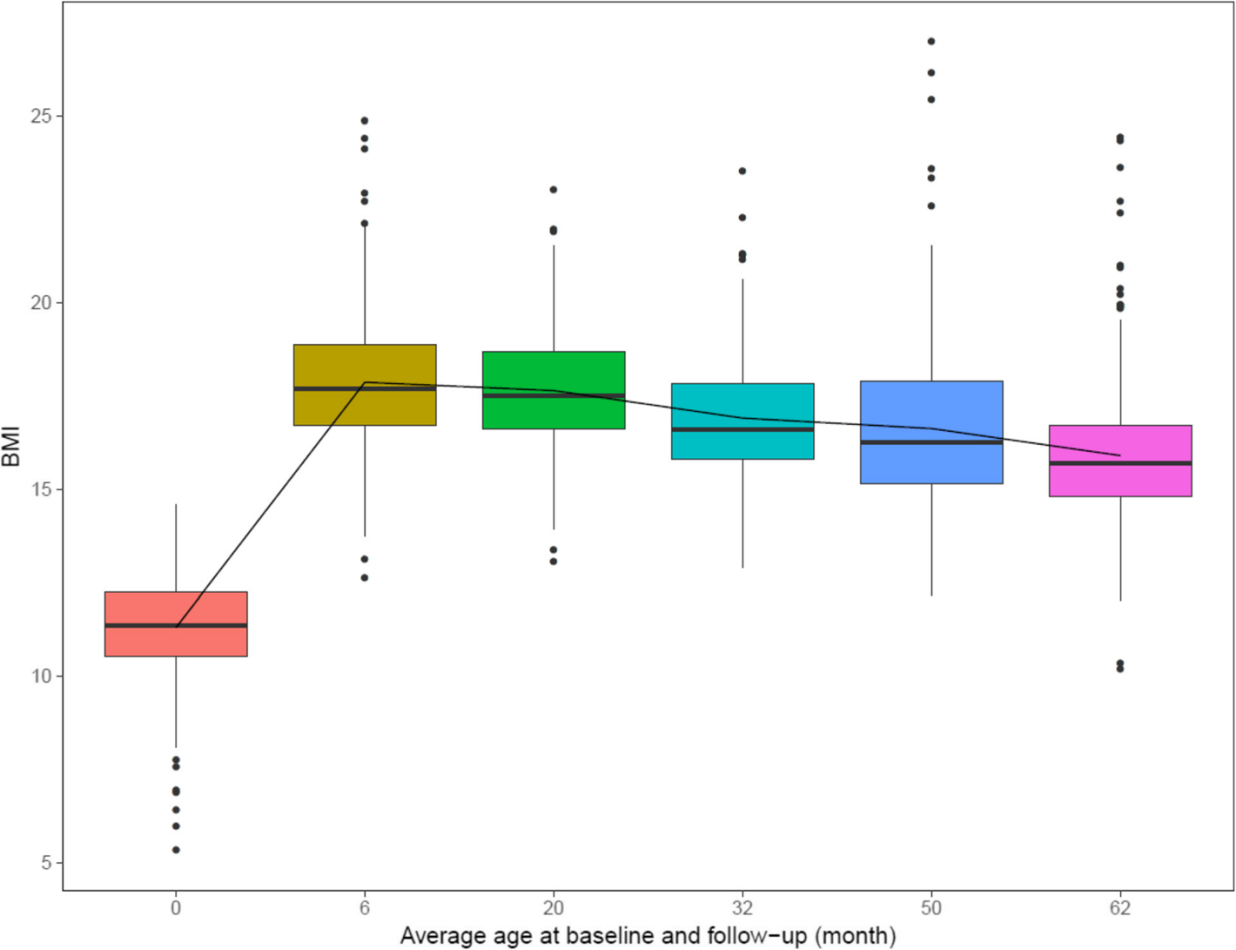
Distributions of BMI and trajectories of average BMI for analyzed QNTS participants. The trajectory of average BMI at each follow-up time point based on the entire analysis sample is highlighted by the black line. Boxplots represent the quartile ranges of BMI at each follow-up time point. Outliers are denoted by black points

Figure 7 shows the BMI distributions of all QNTS individuals included in our analysis at each time point. The trend demonstrated a phase of rapid increase before the first follow-up point (average age = 6 months). This was followed by a plateauing phase of moderate decline in average BMI. When stratified by genetic and environmental variables, some subgroups showed large deviation from the aforementioned overall pattern (See Section S3, Additional file 1). Table 2 displays the descriptive statistics for the distributions of environmental exposure variables and other factors of interest (individual average BMI, age, zygosity and sex). Mean BMI over the follow-up was 15.09 (SD = 1.59). Zygosity was balanced between MZ and DZ twin pairs, but most of the pairs had the same sex. Over half of the individuals were judged to have equal level of physical activity compared to peers. On average, study participants attended daycare and had adequate sleep for around half of the available follow-up time. Only Caucasian individuals were included in the final analysis to control for potential population stratification.

**Table 2:** Characteristics of the analysis sample

	No. of individuals	Proportion or Mean (SD)
Individuals	536	
Individual average BMI	536	15.09 (1.59)
Age		
At birth	536	0 (0)
Follow-up #1	536	6.28 (0.74)
Follow-up #2	531	19.49 (0.75)
Follow-up #3	497	31.77 (0.96)
Follow-up #4	435	49.85 (1.82)
Follow-up #5	478	62.25 (3.23)
Zygosity^a^		
DZ twin	149	0.51
MZ twin	143	0.49
Sex^a^		
Female-female twin	122	0.42
Male-male twin	102	0.35
Female-male twin	68	0.23
Physical Activity		
More	156	0.37
Equal	253	0.59
Less	17	0.04
Individual proportion of follow-up attending daycare facility	530	0.42 (0.37)
Individual proportion of follow-up with sufficient sleep	524	0.57 (0.31)
Race		
Caucasian	518	1
Other	0	0

^a^Analysis conducted with respect to each twin pairs instead of individuals

*No* number, *SD* standard deviation, *BMI* body mass index, *MZ* monozygotic, *DZ* dizygotic

**Fig. 8:**
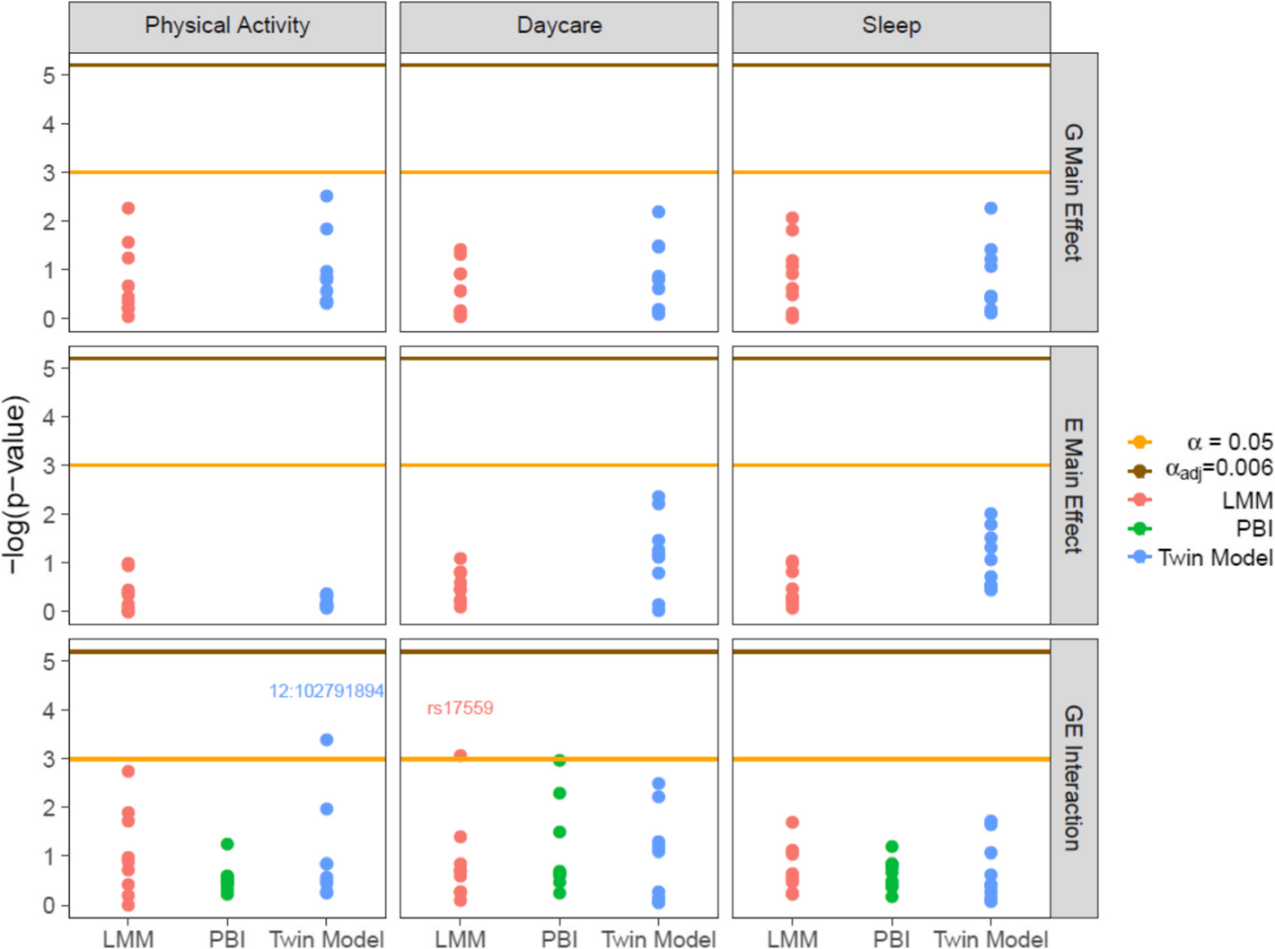
Significance of interactions between the IGF pathway genes and environmental factors. Negative natural log transformed *p*-values from the twin model, the linear mixed model and the PBI test were compared with unadjusted significance level (ɑ = 0.05) and Bonferroni-adjusted level (ɑ_adj_ = 0.006). Genetic (IGF-1, IGFALS) and environmental (physical activities, daycare attendance, sleep duration) main effects were evaluated by the twin and linear mixed models. All methods estimated significance of gene-environment interaction effects. Any effect with significance per unadjusted level (*p*-value < 0.05) is labeled for the involved IGF-1 or IGFALS SNP. LMM = linear mixed model; PBI = partition based score I test

We examined the significance of potential GE interactions as well as each factor’s main effect using the twin model, the linear mixed model and the PBI test. The PBI test evaluated interaction effects only. As shown in Fig. 8, none of the effects were significant after correcting for multiple comparisons among SNPs interacting with the same environmental factor (number of tests = 9). Interactions between physical activity and IGF-1 12:102791894 SNP or between daycare attendance and IGFALS rs17559 SNP were significant before adjustment. In this case, physical activity and IGF-1 12:102791894 interaction was detected by the twin model, while daycare attendance and IGFALS rs17559 interaction was captured by the linear mixed model.

## Discussion

In this study, we assessed the theoretical performance of three longitudinal family methods for detecting GE interactions, and we applied these methods to data from QNTS in order to evaluate the joint effect of the IGF pathway genes (IGF-1, IGFALS) and environmental factors (physical activity, daycare attendance and sleep duration) on childhood obesity. Both the simulation analysis and the QNTS data analysis have produced results highlighting the challenges of modeling complex correlation and interaction relationships. When reasonable approximation of the differences between individual BMI trends was achieved, simplification of longitudinal data structure by averaging the outcomes over different time points did not negatively impact the performance. The analytical models had different detection powers when the GE interactions were non-linear. This pattern of performances suggested that our methods were not robust to interaction model misspecification, and fitting the correct interaction relation in a given model is important to its performance. The analysis of the QNTS data did not reveal any statistically significant GE interactions for the IGF pathway genes after correction for multiple testing. The suggestive interaction between the IGF-1 variant at position 102,791,894 of chromosome 12 and physical activity may serve as hypothesis for further studies.

Longitudinal correlation was modeled by the linear mixed model as this model allows use of all of the available data points from each individual. However, we found that summarizing the repeated outcomes by their mean and applying the cross sectional twin study model had higher power to detect GE interactions. Other studies have also demonstrated good power for detecting genetic main effects using methods that summarize individual longitudinal outcomes with their averages [33, 47]. Mechanistically, the high power of using the averaging method may be attributed to the fact that the mean statistic can retain relevant information while reducing random noise. This is because of its formulation as a weighted sum of data points, the mean statistic can therefore account for the effect from each repeated measure and produce better representation of any systematic pattern. An example to illustrate the importance of this averaging procedure to method performance is contrasting with analysis using only one of the repeated outcomes, which has resulted in lower power [32, 47, 48]. This difference in performance between the two simplification approaches could be due to the fact that when compared to a single time point, using the mean over time was able to preserve more information from the original longitudinal measures while minimizing random noise. At the same time, using mean statistics can simplify the longitudinal correlation structure while retaining important information from the original data set. In this way, averaging repeated outcomes can be an optimal compromise between accuracy and parsimony when modeling longitudinal data, and may contribute to its better performance over the more accurate linear mixed model. Considering these results, it appears that simplifying longitudinal correlation with mean statistics is able to achieve good performance in scenarios with simple longitudinal data pattern. At the same time, it is possible for more complex longitudinal trajectories to occur in nature. Genetic and environmental effects on outcome may be variable across time. In these cases, averaging outcome values across time could lead to loss of information and a decrease in statistical power.

Because of GE interaction’s potentially complex nature, we should consider how much information would be lost if a simple interaction model is imposed on data from a more complex gene-environment relationship. Unlike single factor effects, GE interactions represent multi-dimensional relationships between several factors. The multiplicative term commonly used in regression models to represent interactions will not be reflective of all possible relationships, since it only accurately portrays linear trends. Including additional higher order quadratic interaction terms in the model may capture a specific type of complex GE interactions, but this type of modeling is not always done [49]. In our analysis, the performance of regression approaches (the twin model and the linear mixed model) dropped whenever the interaction scenario was not linear. This suggested that the erroneous assumption of a linear relationship was not reasonable, and may result in loss of power when imposed on a non-linear scenario.

In addition to the loss of power observed for regression methods under non-linear interactions, the nonparametric PBI test also had low robustness to different interaction relations. Lower detection power was observed for the PBI test when the underlying interaction model differed from its best-case scenario (XOR interaction). This means that the performances of all three methods tested in this study were sensitive to changing interaction relations in the data. Specialized methods may be needed for specific interaction scenarios in order to achieve adequate detection power.

An interaction that was significant before adjustment was between a variant in IGF-1 at chromosome 12 position 102,791,894 and physical activity. It was detected by the twin model (*p* = 0.034) and had near significant *p*-value under the linear mixed model (0.065). This SNP was not documented in the dbSNP database, and is positioned outside the protein coding region of the IGF-1 gene. If there is an actual interaction effect, the variant may influence IGF-1 through gene regulation mechanisms. The effect of physical activity on the IGF pathway may be non-linear, since there is evidence for a threshold-dependent pattern of stimulation on the GH signaling by physical activity [19, 20]. If physical activity interacts with the IGF-1 gene in a similar manner, then the joint effect pattern may not be adequately captured with the methods used in this study. Thus, further characterization of this variant and its interplay with physical activity is required before making any concrete conclusions.

Our study had limitations that should be noted when considering the result. For simulation scenarios, we made several simplifying assumptions that could potentially limit the generalizability of our findings. For example, the environmental exposure and the genetic effect were simulated to be either constant or monotone changes over time. In reality, it is possible for more complex longitudinal trajectories. In this study, we focused our simulation design effort on the variability of the GE interaction relationships. We had considered several representative and plausible interaction relationships that might be encountered in nature. Scenarios such as the XOR relationships are not commonly examined in the context of GE interactions. The result of our study highlighted the poor robustness of the studied methods to non-linear interactions and could serve as starting point for future investigations with different GE interaction or longitudinal effect patterns. The analysis of the actual QNTS data was also limited by data constraints. For example, environmental exposure data were collected using interview questionnaires. Potential measurement error on environmental factors due to the usage of interview questionnaire can influence statistical power to detect GE interactions. Wong et al. (2003) showed that studies with repeated and precise measurements of exposure and outcome variables can have as much powerful to detect GE interactions as studies with 20 times the sample size [50]. Thus, the statistical power of our study may be limited by its moderate sample size and potentially imprecise measurements of environmental exposures. Because of the gaps in the available data and lack of adequate statistical models, we were also not able to assess the environmental effects as time-varying. At the same time, the analysis procedure taken by this study has low potential for significant selection bias, as our analysis sample was not statistically different from the whole QNTS data (See Section S1 Table S1.1, Additional file 1). Population stratification was also controlled via restricting analysis to Caucasian individuals. Thus the results should be treated as exploratory in nature, and the putative GE interaction between the IGF-1 variant and physical activity represents an interesting hypothesis that warrants further investigation.

## Conclusion

Using longitudinal family studies to investigate GE interactions holds great potential for increasing our understanding of childhood obesity etiology. However, when analyzing longitudinal family data, the methodological issues raised in this study should be considered. Although simplifying the over time repeated outcomes with their averages worked fairly well, all of the methods considered in this study were not robust to misspecification of the interaction relationship. This highlights the need for more robust methods when studying GE interactions in the longitudinal family context. Equally important, characterization of how different GE interaction relationships influence method performance is also needed. Future study design should give more consideration to the inherent complexities of GE interactions. These lessons could be applied when performing further studies on the putative interaction between the IGF-1 variant and physical activity, as suggested by our result.

## Additional file

Additional file 1: Supplementary Information and Results. Appendix S1 contains additional information on 1) Constructing kinship matrix when fitting linear mixed model; 2) Data quality control filtering process overview; 3) Deriving the daily sleep time data and the American Academy of Sleep Medicine guideline; 4) Minor allelic frequencies of IGF-1 and IGFALS SNPs. Appendix S2 contains 1) Results for the linear mixed model and twin model with co-dominant genetic effect coding; 2) Behaviour of the PBI Test during simulation analysis. Appendix S3 contains trajectories of average BMI at each follow-up time point. (DOCX 1030 kb)
