# Supplementary material for: Modeling gene-environment interactions in longitudinal family studies: a comparison of methods and their application to the association between the IGF pathway and childhood obesity
Source: BMC Med Genet. 2019 Jan 11;20:9. doi: 10.1186/s12881-018-0739-x (PMC6329142; doi:10.1186/s12881-018-0739-x)
Supplement: Supplementary file 1 — Supplementary Information and Results. Appendix S1 contains additional information on 1) Constructing kinship matrix when fitting linear mixed model; 2) Data quality control filtering process overview; 3) Deriving the daily sleep time data and the American Academy of Sleep Medicine guideline; 4) Minor allelic frequencies of IGF-1 and IGFALS SNPs. Appendix S2 contains 1) Results for the linear mixed model and twin model with co-dominant genetic effect coding; 2) Behaviour of the PBI Test during simulation analysis. Appendix S3 contains trajectories of average BMI at each follow-up time point. (DOCX 1030 kb) [file 12881_2018_739_MOESM1_ESM.docx]

Table of Contents

[Appendix S1 2](#_Toc527547149)

[Constructing kinship matrix when fitting linear mixed model 2](#_Toc527547150)

[Data quality control filtering process overview 4](#_Toc527547151)

[Deriving the daily sleep time data and the American Academy of Sleep Medicine guideline 9](#_Toc527547152)

[Minor allele frequencies of IGF-1 and IGFALS SNPs 10](#_Toc527547153)

[Appendix S2 11](#_Toc527547154)

[Results for the linear mixed model and twin model with co-dominant genetic effect coding 11](#_Toc527547155)

[Behaviour of the PBI Test during simulation analysis 15](#_Toc527547156)

[Appendix S3 20](#_Toc527547157)

[Trajectories of average BMI at each follow-up time point 20](#_Toc527547158)

# Appendix S1

## Constructing kinship matrix when fitting linear mixed model

Given $n$ individuals, we construct a $n\times n$ kinship matrix with kinship coefficient entries denoted by $\varphi_{ij}$, where $i=1,2\ldots n$ and $j=1,2\ldots n$. For individuals with his/herself (ie. diagonal entries, $i=j$), the kinship coefficient $\varphi_{ij}$ will equal 0.5. If two individuals are from the same MZ twin pair, then $\varphi_{ij}=0.5$, whereas for DZ twin individuals $\varphi_{ij}=0.25$. When two individuals come from separate twin pairs, they are assumed to be unrelated so $\varphi_{ij}=0$. An example matrix is as follow:

|  | DZ twin 1 | DZ twin 2 | MZ twin 1 | MZ twin 2 |
| --- | --- | --- | --- | --- |
| DZ twin 1 | 0.5 | 0.25 | 0 | 0 |
| DZ twin 2 | 0.25 | 0.5 | 0 | 0 |
| MZ twin 1 | 0 | 0 | 0.5 | 0.5 |
| MZ twin 2 | 0 | 0 | 0.5 | 0.5 |

The kinship coefficients are now described in more detail. Mathematically, the kinship coefficient represents the probability of obtaining identical alleles when one allele at a given genetic locus is randomly sampled from two individuals. Thus, when randomly sampling alleles from a single individual, the probability of obtaining the same allele twice is 0.5. Since MZ twins share identical genomic DNA, the kinship coefficient between the pair is also 0.5. DZ twins on average share half of their genomic DNA from the same ancestor, so the probability of sampling the same allele between DZ individuals is 0.25. Assuming all twin pairs coming from independent families, the probability of sampling the same allele between any individuals from two families is 0.

To facilitate model fitting, a close approximation of the kinship matrix was obtained to ensure the matrix was positive definite. In this approximated matrix, the kinship coefficient between the two individuals of a MZ twin pair was 0.49999999 instead of 0.5, while the coefficients for all other relationships remained the same.

Data quality control filtering process overview

The initial study sample before any imputations and exclusions (including those based on ethnicity and missing BMI data) contained 558 individuals with both sequencing and environmental data. Figure S1.1 shows the subsequent quality control steps to obtain the final study sample. Out of the initial study sample, 33 and 57 individuals were excluded for having >10% missing rate for the IGF-1 and IGFALS sequencing data, respectively. MZ individuals (195 for IGF-1 and 190 for IGFALS) without sequencing data were later imputed based on their siblings’ genotypes assuming MZ twins share identical DNA. After merging the imputed sequencing data with environment data, 682 individuals had data on both genetic and environment variables. 99 individuals were then excluded for having more than 4 missing entries for BMI. To control for population stratification, 47 individuals with non-Caucasian ethnicity were excluded. The final sample used for analyses consisted of 536 individuals from 292 QNTS families. 244 of the 292 families had data on both twin individuals. 48 families had data on only 1 individual. 143 families were MZ twins, and 149 families were DZ twins.

Figure S1.1. Data quality control steps to obtain the final sample for analyses

Initial study sample consisted of individuals with both sequencing and environment data. An intermediate sample was obtained after filtering out individuals with >10% missing sequencing data and imputing data for monozygotic (MZ) twin individuals. Final study sample was selected after excluding individuals with >4 missing BMI measures and/or those whose ethnicity was not Caucasian.

Table S1.1 compares the distributions of variables in the final sample used for analyses with those from the whole QNTS data. Overall, the two samples were similar with no significant difference except individual BMI average, age at follow-up point 4 (average age = 50 months) and race. The individual BMI averages were higher in the analysis sample (mean (SD): 15.09 (1.59) vs. 14.8 (2.06)). Mean age at follow-up point 4 was lower for the analysis sample (49.85 (1.82) vs. 50.08 (1.89)). Difference in ethnic composition was expected since we excluded all non-Caucasian individuals from the analysis.

Table S1.1. Distribution characteristics and comparisons between the analysis sample and the available data

|  |  | **Analyzed individuals** | |  | **Available QNTS individuals^1^** | |  |
| --- | --- | --- | --- | --- | --- | --- | --- |
|  |  | **No. of individuals** | **Proportion or Mean (SD)** |  | **No. of individuals** | **Proportion or Mean (SD)** |  |
| Individuals |  | 536 |  |  | 810 |  |  |
| BMI |  |  |  |  |  |  |  |
| At birth |  | 499 | 11.3 (1.37) |  | 698 | 11.25 (1.41) |  |
| Follow-up #1 |  | 344 | 17.86 (2.04) |  | 436 | 17.96 (2) |  |
| Follow-up #2 |  | 182 | 18.05 (3.17) |  | 234 | 18.06 (2.89) |  |
| Follow-up #3 |  | 120 | 16.91 (1.91) |  | 172 | 16.87 (1.86) |  |
| Follow-up #4 |  | 117 | 16.63 (2.63) |  | 156 | 16.64 (2.5) |  |
| Follow-up #5 |  | 453 | 15.901 (1.72) |  | 599 | 15.899 (1.7) |  |
| Individual average BMI |  | 536 | 15.09 (1.59) |  | 795 | 14.8 (2.06) | * |
| Age |  |  |  |  |  |  |  |
| At birth |  | 536 | 0 (0) |  | 810 | 0 (0) |  |
| Follow-up #1 |  | 536 | 6.28 (0.74) |  | 802 | 6.29 (0.74) |  |
| Follow-up #2 |  | 531 | 19.49 (0.75) |  | 782 | 19.53 (0.81) |  |
| Follow-up #3 |  | 497 | 31.77 (0.96) |  | 734 | 31.85 (1.02) |  |
| Follow-up #4 |  | 435 | 49.85 (1.82) |  | 610 | 50.08 (1.89) | * |
| Follow-up #5 |  | 478 | 62.25 (3.23) |  | 638 | 61.98 (3.12) |  |
| Zygosity^2^ |  |  |  |  |  |  |  |
| DZ twin |  | 149 | 0.51 |  | 197 | 0.49 |  |
| MZ twin |  | 143 | 0.49 |  | 208 | 0.51 |  |
| Sex^2^ |  |  |  |  |  |  |  |
| Female-female twin |  | 122 | 0.42 |  | 164 | 0.405 |  |
| Male-male twin |  | 102 | 0.35 |  | 168 | 0.415 |  |
| Female-male twin |  | 68 | 0.23 |  | 73 | 0.18 |  |
| Physical Activity |  |  |  |  |  |  |  |
| More |  | 156 | 0.37 |  | 254 | 0.40 |  |
| Equal |  | 253 | 0.59 |  | 354 | 0.57 |  |
| Less |  | 17 | 0.04 |  | 21 | 0.03 |  |
| Individual proportion of follow-up attending daycare facility |  | 530 | 0.42 (0.37) |  | 782 | 0.41 (0.38) |  |
| Individual proportion of follow-up with  sufficient sleep |  | 524 | 0.57 (0.31) |  | 764 | 0.54 (0.32) |  |
| Race |  |  |  |  |  |  | * |
| Caucasian |  | 518 | 1 |  | 704 | 0.91 |  |
| Other |  | 0 | 0 |  | 72 | 0.09 |  |

^1^Analysis sample compared to individuals with available BMI and environment exposure data.

^2^Analysis performed with respect to twin pairs instead of individuals.

*Significant difference p<0.05 with ANOVA test or Chi-squared test where appropriate. Test p-value did not account for clustering, which will be more conservative if adjusted.

## Deriving the daily sleep time data and the American Academy of Sleep Medicine guideline

For daytime sleep, the categories were “less than 1 hr”, “1-2 hr”, “2-3 hr”, “3-4 hr” and “more than 4 hr”. For night sleep, the response levels consisted of “less than 4 hr”, “4-5 hr”, “5-6 hr”… and so on till “9-10 hr”, “more than 10 hr” and “more than 8 hr”. We first converted categorical responses to numerical values. Two categories “does not sleep 5 hr straight” and “sleep 5 hr straight” were excluded from our analysis since they only appeared in the first follow-up point (6 month), and were not meaningful for conversion into numerical values. For all other responses, we converted time categories into continuous hour data by selecting the median at each level when possible. For example, “1-2 hr” was coded as 1.5 hours of sleep. For categories such as “less than 1 hr” or “more than 8hr”, we coded using the implied upper and lower bound (1 and 8 hours) respectively. Once converted into numerical figures, we calculated the daily sleep time for each individual by adding the daytime and night sleep hours. Individual daily sleep duration was then compared with the minimum sleep time recommendation from the American Academy of Sleep Medicine (Table S1.2), and scored as “1” for meeting the guideline requirement or “0” otherwise.

Table S1.2. Minimum recommended sleep time by the American Academy of Sleep Medicine

| Age (month) | Minimum Sleep Time (hours) |
| --- | --- |
| 4-11 | 12 |
| 12-35 | 11 |
| 36-71 | 10 |
| 72-155 | 9 |

## Minor allele frequencies of IGF-1 and IGFALS SNPs

Comparison of MAF for the analyzed genetic loci between Caucasian individuals and non-Caucasian individuals are summarized in Table S1.3. The allelic distributions at several loci were significantly different (IGF-1: rs6219, 12:102791894, rs6214 and IGFALS: rs17559, rs3751893). Hence, we adjusted for potential confounding effect due to population stratification by excluding non-Caucasian individuals.

Table S1.3. Description of analyzed genetic loci and their allele frequencies

| **SNP** |  | **Genomic Position (chr:bp)** |  | **Gene** |  | **Allele** |  | **MAF** | |  |
| --- | --- | --- | --- | --- | --- | --- | --- | --- | --- | --- |
|  |  |  |  |  |  |  |  | **Race** | |  |
|  |  |  |  |  |  | **(Other : Minor)** |  | **Caucasian** | **Other** |  |
| rs6219 |  | 12:102396414 |  | IGF-1 |  | C:T |  | 0.07 | 0.17 | * |
| rs1140655 |  | 12:102397002 |  | IGF-1 |  | T:A |  | 0.30 | 0.37 |  |
| rs207473318 |  | 12:102791864 |  | IGF-1 |  | T:A |  | 0.10 | 0.11 |  |
| 12:102791894** |  | 12:102791894 |  | IGF-1 |  | C:A |  | 0.06 | 0.02 | * |
| rs6214 |  | 12:102399791 |  | IGF-1 |  | C:T |  | 0.46 | 0.35 | * |
| rs6220 |  | 12:102400737 |  | IGF-1 |  | A:G |  | 0.27 | 0.33 |  |
| rs28399924 |  | 12:102402246 |  | IGF-1 |  | T:A |  | 0.17 | 0.15 |  |
| rs17559 |  | 16:1791032 |  | IGFALS |  | G:A |  | 0.10 | 0.23 | * |
| rs3751893 |  | 16:1792208 |  | IGFALS |  | G:A |  | 0.18 | 0.31 | * |
| MAF: minor allelic frequency; chr: chromosome; bp: basepair | | | | | | |  |  |  |  |
| *Significant difference p<0.05 with Fisher's exact test (with simulation approximated p-value) | | | | | | | | | | |
| **SNP not identified in dbSNP (build 148) | | | | |  |  |  |  |  |  |

# Appendix S2

## Results for the linear mixed model and twin model with co-dominant genetic effect coding


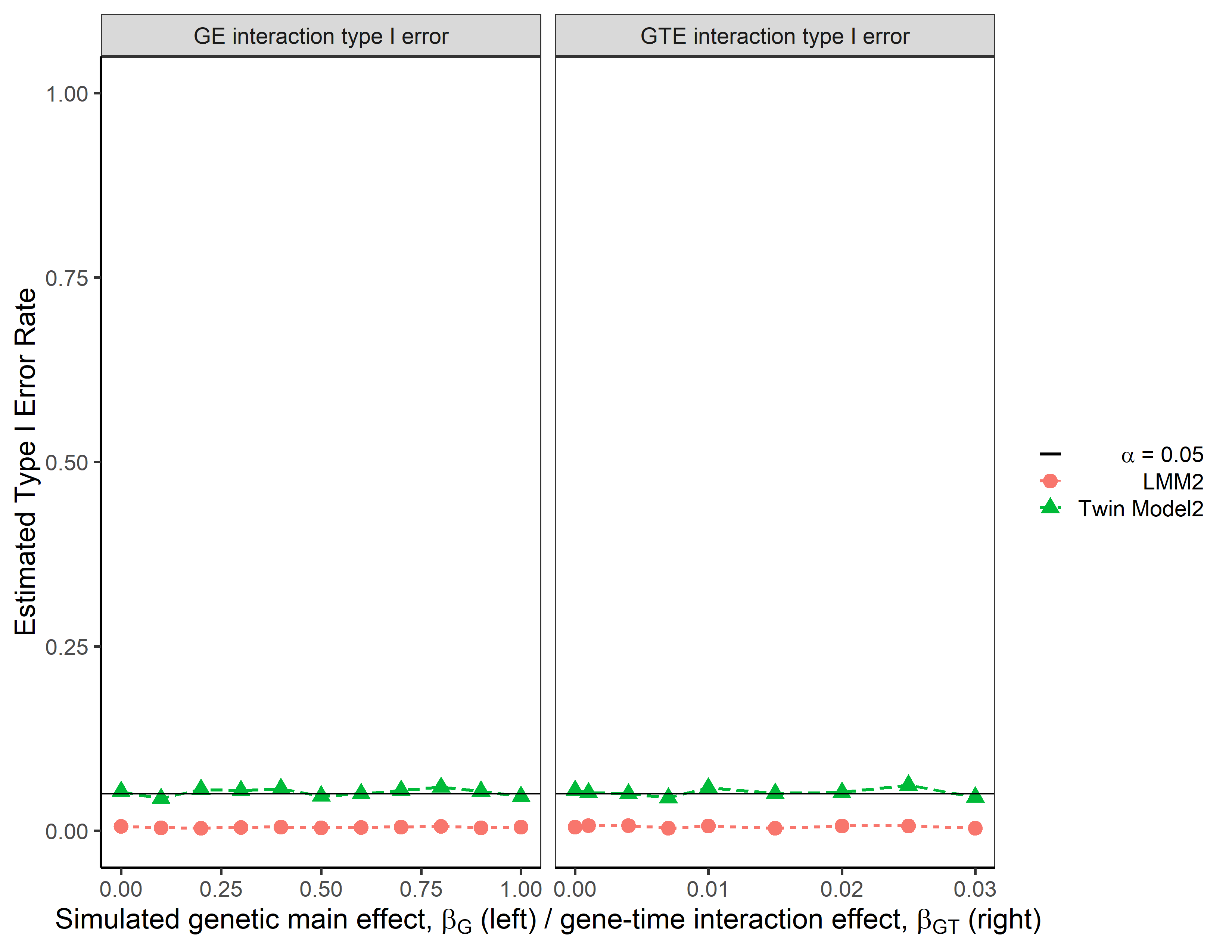

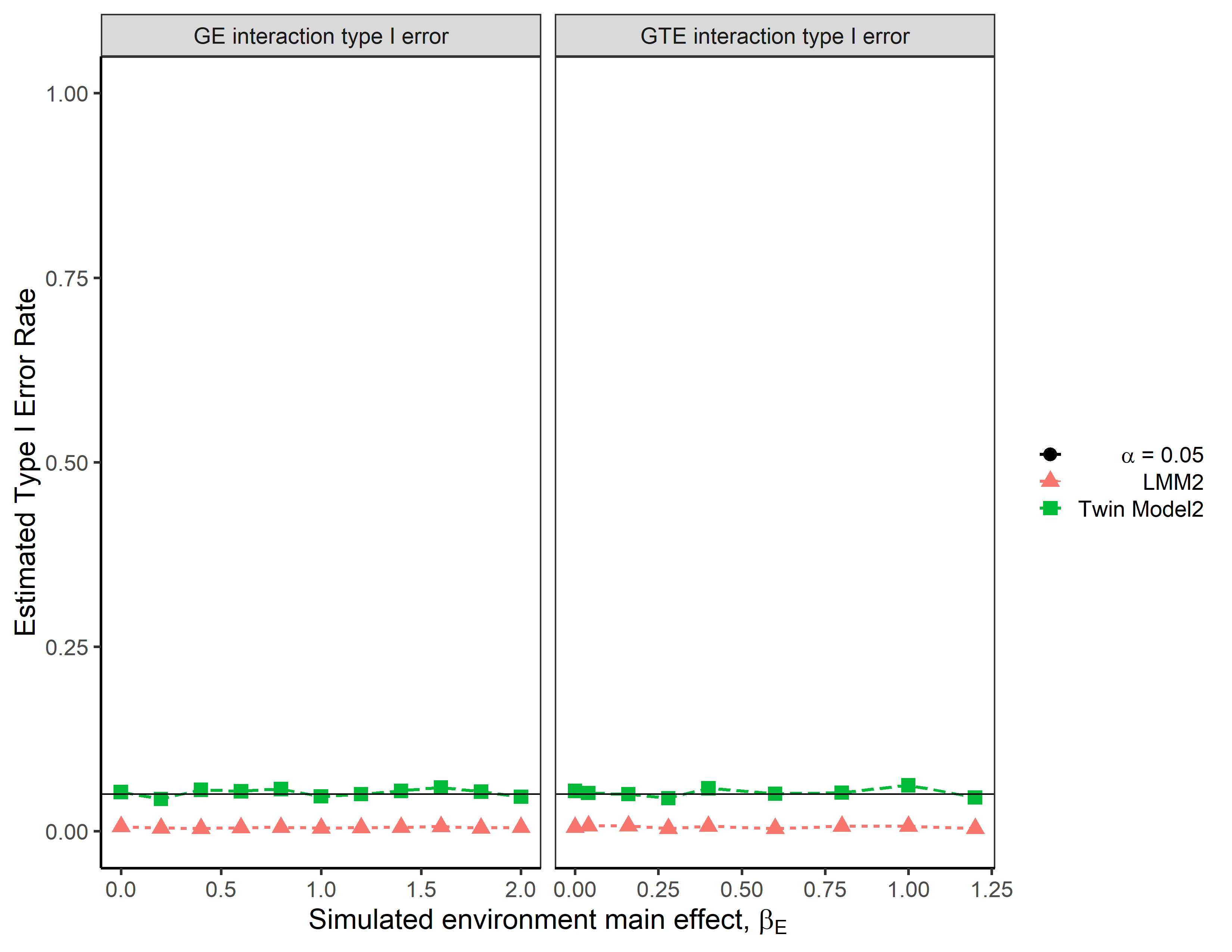


Figure S2.1. Estimated type I error rates for the compared analytical approaches

Type I error rates were estimated for each analytical approach as the proportion of false positive results (p-value < 0.05) calculated over 2000 simulation replicates with no interaction effect. Estimated type I error rates were plotted for GE interaction (includes a genetic main effect, left panel) and GTE interaction (includes a gene-time interaction effect, right panel). The environmental main effect ($\beta_{E}$) was included for all null scenarios. GE = gene-environment; GTE = gene-time-environment; LMM2 = linear mixed model with co-dominant genetic effect coding; Twin Model2 = twin model with co-dominant genetic effect coding.


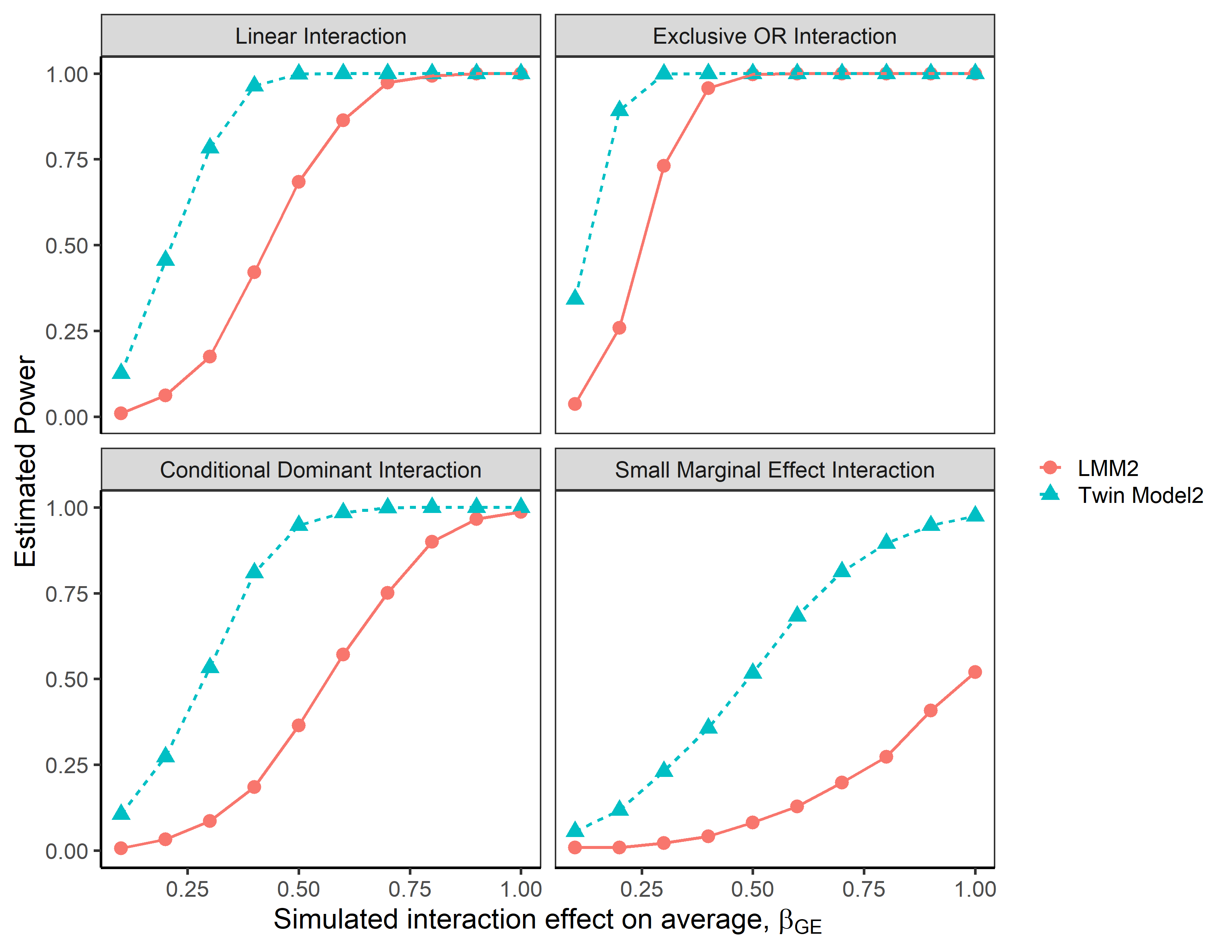


Figure S2.2. Estimated power to detect GE interaction effect on the average scenarios

Power was estimated for each analytical approach as the proportion of true positive result (p-value < 0.05) over 2000 simulation replicates for the considered interaction scenarios. LMM2 = linear mixed model with co-dominant genetic effect coding; Twin Model2 = twin model with co-dominant genetic effect coding.


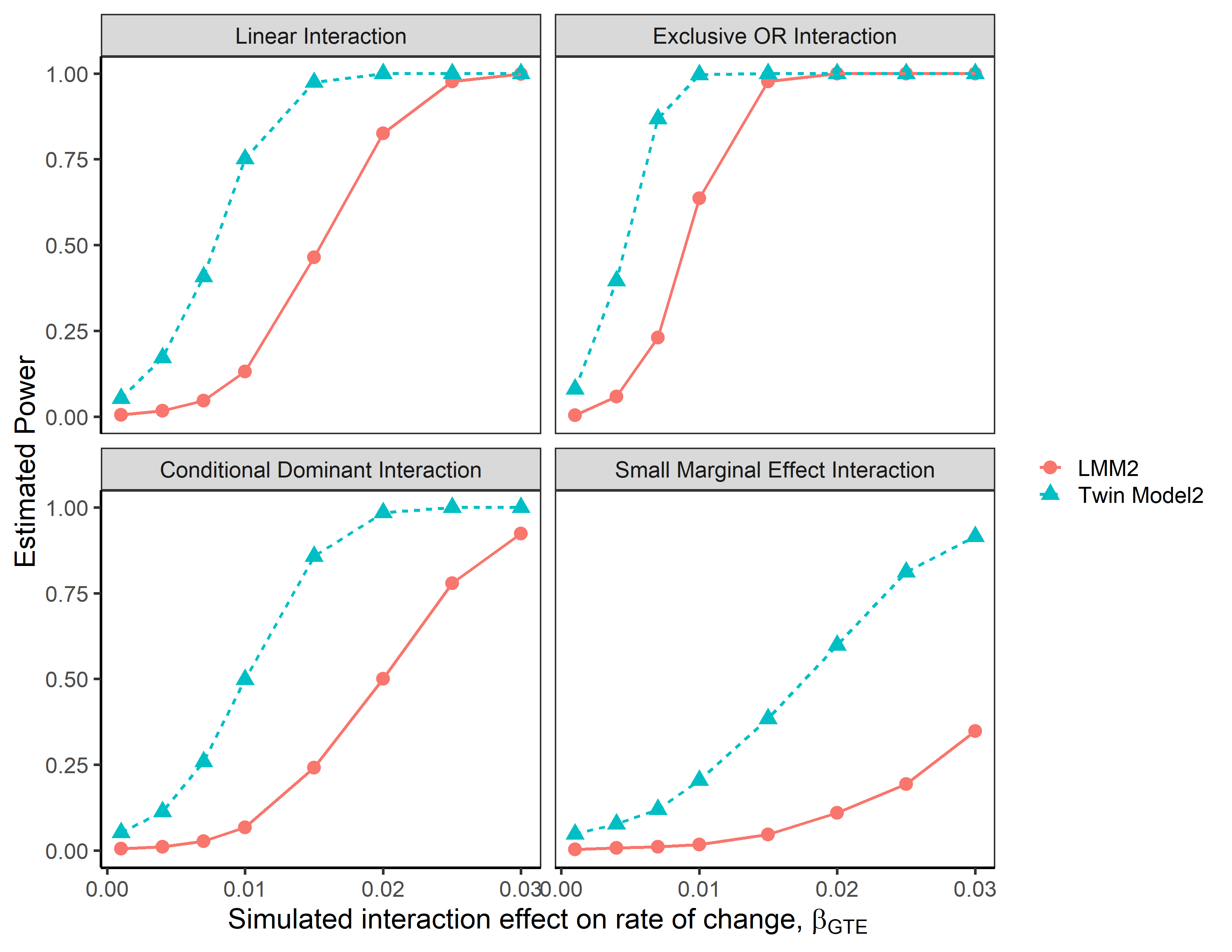


Figure S2.3. Estimated power to detect GTE interaction effect on the rate of change over time scenarios

Power was estimated for each analytical approach as the proportion of true positive result (p-value < 0.05) over 2000 simulation replicates for the considered interaction scenarios. LMM2 = linear mixed model with co-dominant genetic effect coding; Twin Model2 = twin model with co-dominant genetic effect coding.

## Behaviour of the PBI Test during simulation analysis

From our simulation analysis, we observed unusual behavior for the PBI test. The test was either too conservative or very prone to false positives under different scenarios. Since the PBI test statistic is dependent on the dispersion statistic values (I_GE_, I_E_ and E_G_) calculated for different ways of partitioning the dataset, we monitored the values of those statistics for different simulation parameter values. For the no interaction effect scenarios (Figure S2.3), the test statistic PBI decreased steadily as the simulated main effects increased under the null GE interaction scenario. The decrease in the test statistic was due to higher dispersion statistic values when partitioning data by the environmental factor alone, compared to partitioning by both gene-environment factors. This meant that partitioning based on environmental factor level alone appeared to explain more BMI variations. However, in the null GTE interaction scenario, the PBI test statistic remained close to zero regardless of the changes in the effect sizes for GT interaction and the environmental factor. In this case, the dispersion statistic values from gene-environment partitioning and environment alone partitioning were similar. So partitioning data by environmental factor alone or gene-environmental factors yielded similar level of outcome explanatory power.


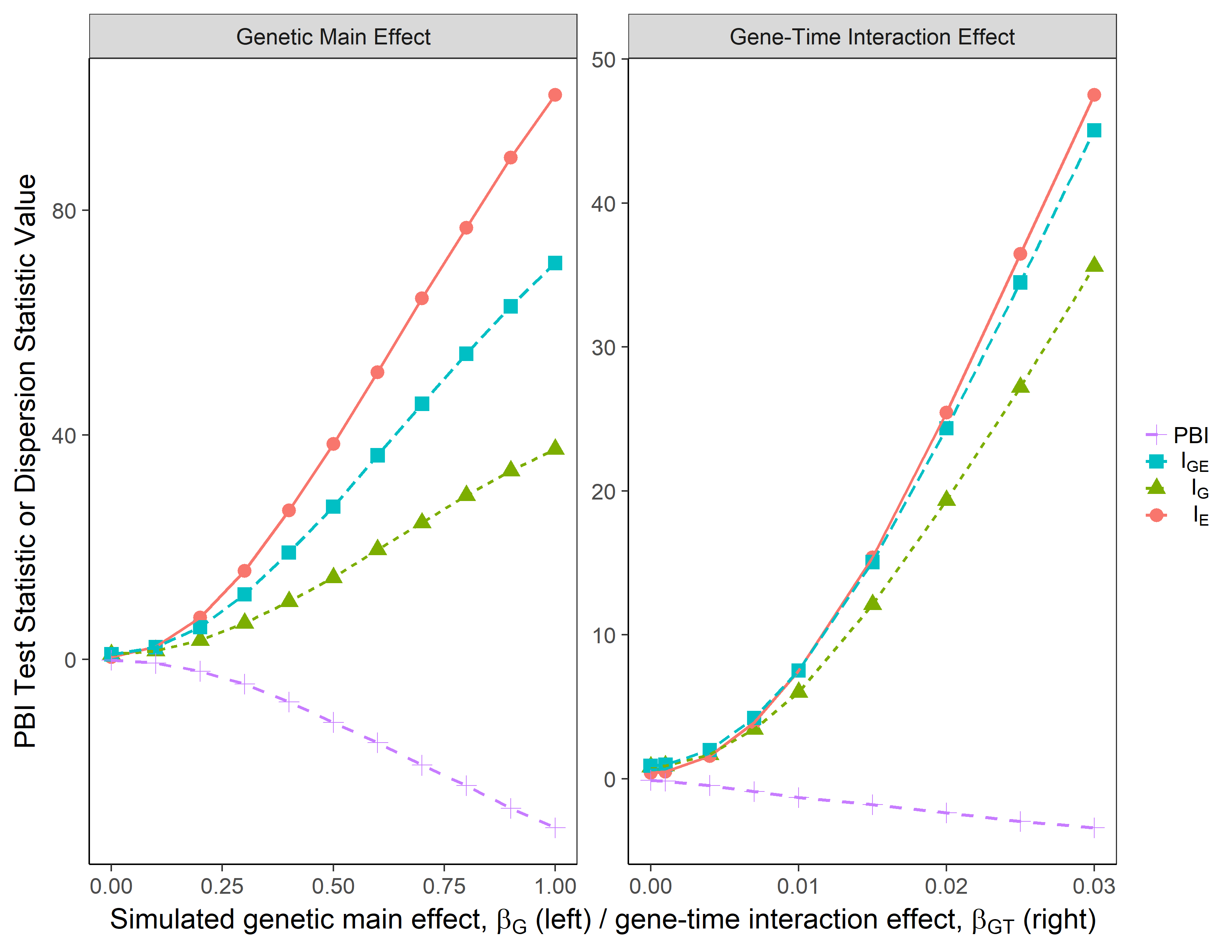

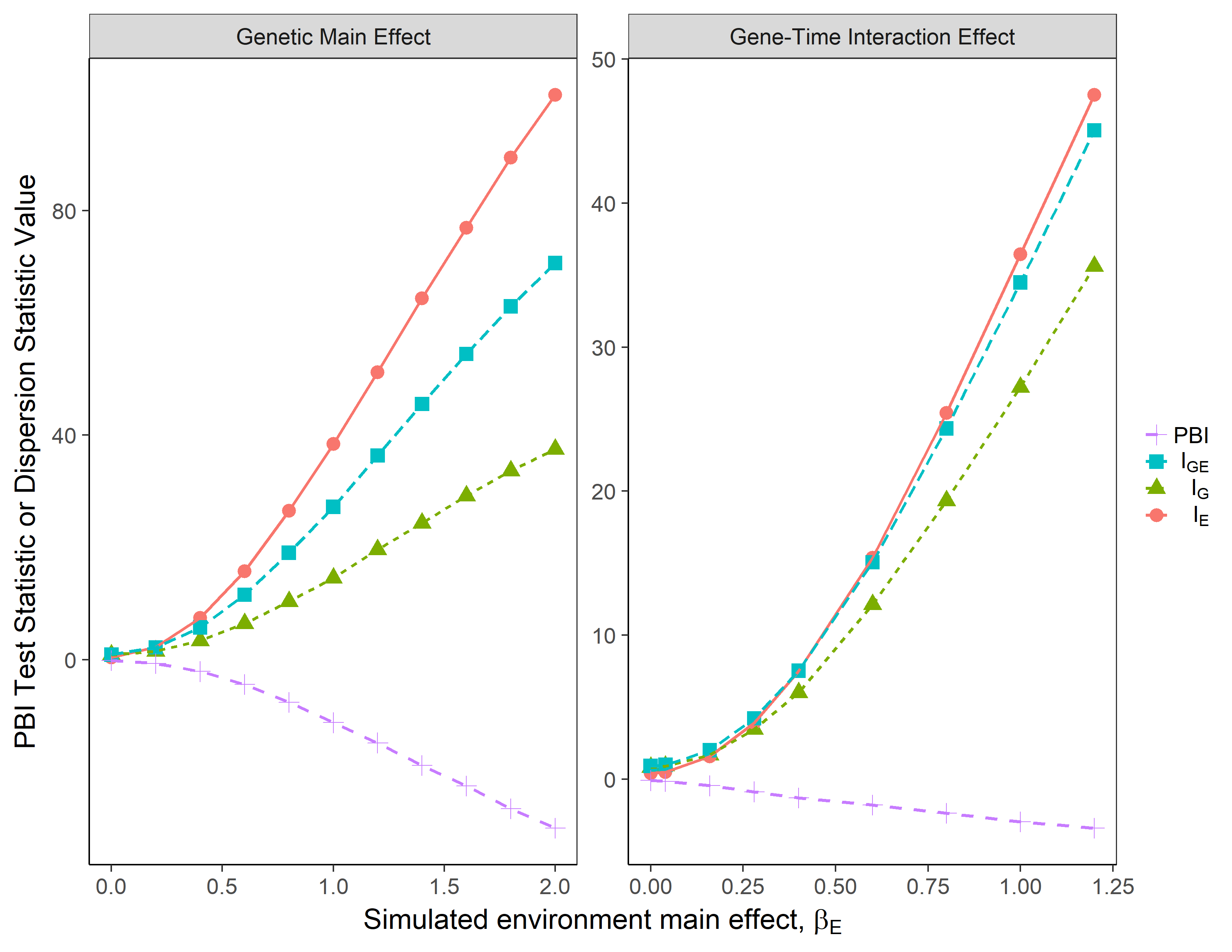


Figure S2.4. Behavior of partition based score I (PBI) test statistics and dispersion statistics for no interaction effect scenarios

Average value over 2000 replicates for PBI test statistics (PBI) and dispersion statistics for gene-environment (I_GE_), gene only (I_G_) and environment only (I_E_) dataset partitioning schemes are plotted. PBI test statistics is equal to the difference between I_GE_ and the maximum of I_G_ or I_E_. The genetic main effect ($\beta_{G}$) and gene-time interaction effect ($\beta_{GT}$) were varied separately for two sets of no interaction scenarios, while the environmental main effect ($\beta_{E}$) was varied for all null scenarios.

Similar behaviour for the PBI test statistics and the dispersion statistics were also observed for simulated GE interaction scenarios (Figure S2.4). For linear and conditional dominant scenarios where the PBI test performed poorly, the PBI test statistics decreased as the GE interaction effect sizes increased. In those scenarios, partitioning based on environmental or genetic factor alone appeared to explain the outcome variation well. This was demonstrated by the higher dispersion statistic values for those partition schemes. For XOR interaction, gene-environment factor partitioning of the dataset consistently explained the outcome better than partitioning based on any single factor. This was reflected by the increasing trend in the test statistic value as well as higher dispersion statistic values from gene-environment partitioning. In the case of a small marginal effect interaction, it was similar to the null GTE interaction scenario. The explanatory powers from partitioning data by gene and by gene-environment factors were similar and so the PBI test statistic remained fairly close to zero. For GTE interaction scenarios where the GE effect was on the rate of change, similar patterns in PBI test statistics and the dispersion statistics were observed (Figure S2.5).


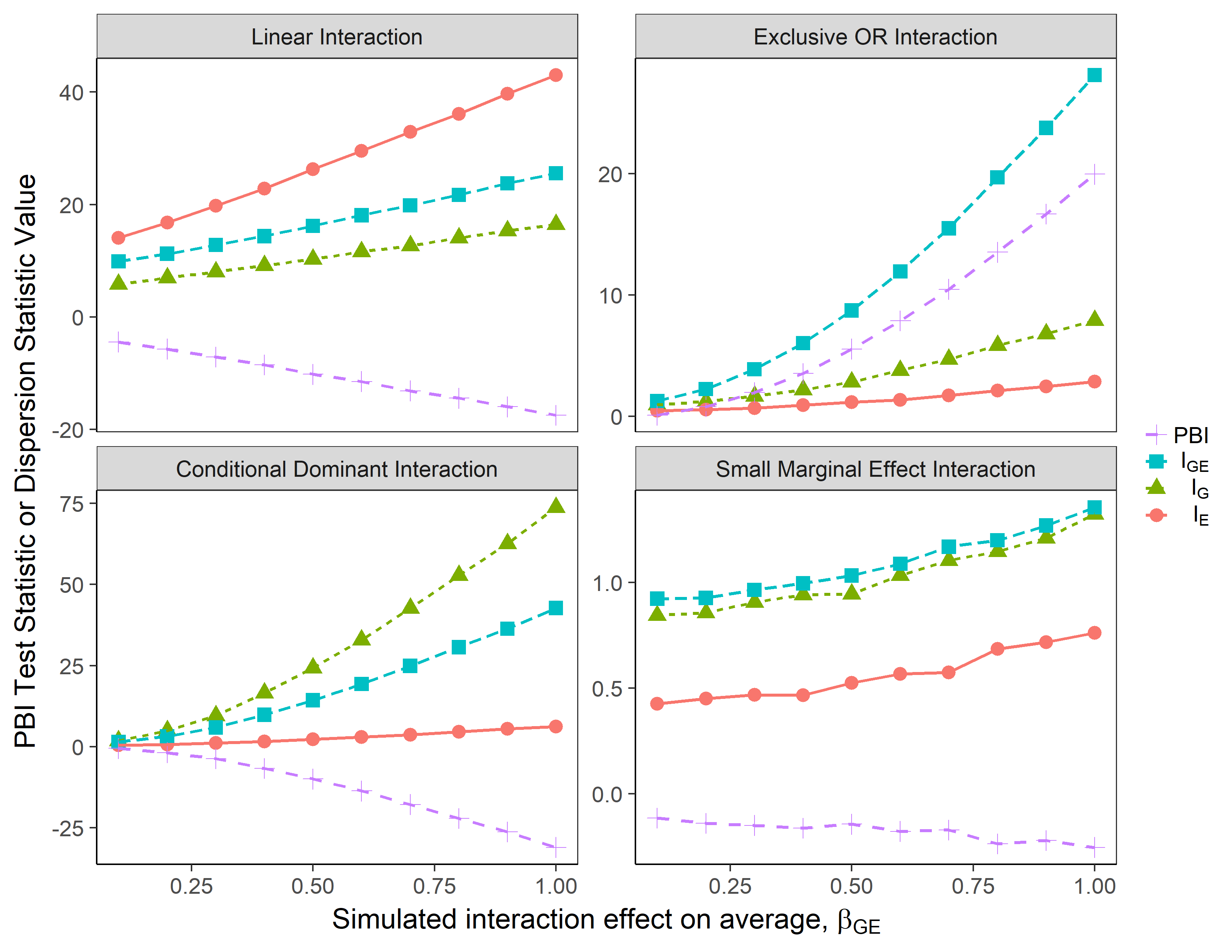


Figure S2.5. Behavior of partition based score I (PBI) test statistics and dispersion statistics for interaction effect on average scenarios

Average value over 2000 replicates for PBI test statistics (PBI) and dispersion statistics for gene-environment (I_GE_), gene only (I_G_) and environment only (I_E_) partitioning schemes are plotted. PBI test statistics is equal to the difference between I_GE_ and the maximum of I_G_ or I_E_. Effect sizes for gene-environment interaction ($\beta_{GE}$) were varied under each interaction relationship.

Figure S2.6. Behavior of partition based score I (PBI) test statistics and dispersion statistics for interaction effect on temporal rate of change scenarios

Average value over 2000 replicates for PBI test statistics (PBI) and dispersion statistics for gene-environment (I_GE_), gene only (I_G_) and environment only (I_E_) partitioning schemes are plotted. PBI test statistics is equal to the difference between I_GE_ and the maximum of I_G_ or I_E_. Effect size for gene-time-environment interaction ($\beta_{GTE}$) were varied under each interactive relationship.

# Appendix S3

## Trajectories of average BMI at each follow-up time point

Figure S3.1. Trajectories of average BMI for analyzed QNTS participants by gene-environment pair

Trajectories of average BMI at each follow-up time point grouped by genetic (G) and environmental (E) factor levels. Panels correspond to the specific pair of the genetic (columns) and the environmental factor (rows). Genetic variables coded as 0, 1 and 2 for the number of minor alleles. Physical activity coded as 0, 1, and 2 being “more”, “equal or “less” physically active compare to peers. Daycare attendance and sleep duration coded as 0, 1, 2 and 3 for the 1st, 2nd, 3rd and 4th quartiles of the continuous proportion measures (see section 2.3.3.1 for more detail on daycare and sleep data definition).

Figure S3.2. Trajectories of average BMI for analyzed QNTS participants by gene-environment pair (continued)

Trajectories of average BMI at each follow-up time point grouped by genetic (G) and environmental (E) factor levels. Panels correspond to the specific pair of the genetic (columns) and the environmental factor (rows). Genetic variables coded as 0, 1 and 2 for the number of minor alleles. Physical activity coded as 0, 1, and 2 being “more”, “equal or “less” physically active compare to peers. Daycare attendance and sleep duration coded as 0, 1, 2 and 3 for the 1st, 2nd, 3rd and 4th quartiles of the continuous proportion measures (see section 2.3.3.1 for more detail on daycare and sleep data definition).
